# Increased Proportion of Fiber-Degrading Microbes and Enhanced Cecum Development Jointly Promote Host To Digest Appropriate High-Fiber Diets

**DOI:** 10.1128/msystems.00937-22

**Published:** 2022-12-13

**Authors:** Guang Pu, Liming Hou, Taoran Du, Wuduo Zhou, Chenxi Liu, Peipei Niu, Chengwu Wu, Wenbin Bao, Ruihua Huang, Pinghua Li

**Affiliations:** a Key Laboratory of Pig Genetic Resources Evaluation and Utilization (Nanjing), Ministry of Agriculture and Rural Affairs, Institute of Swine Science, College of Animal Science and Technology, Nanjing Agricultural University, Nanjing, China; b Industrial Technology System Integration Innovation Center of Jiangsu Modern Agriculture (Pig), Nanjing, China; c Laboratory of Intestinal Microbiology, Huaian Academy, Nanjing Agricultural University, Nanjing, China; d Key Laboratory for Animal Genetics, Breeding, Reproduction and Molecular Design, College of Animal Science and Technology, Yangzhou University, Yangzhou, China; University of California, San Francisco

**Keywords:** high-fiber diet, intestinal microbiota, cecal morphometrics, shotgun metagenomic sequencing, glycoside hydrolases

## Abstract

Previous study found that appropriate high-fiber diet (containing 19.10% total dietary fiber [TDF], treatment II) did not reduce apparent fiber digestibility of Chinese Suhuai finishing pigs and increased the yield of short-chain fatty acids (SCFAs), but too high-fiber diet (containing 24.11% TDF, treatment IV) significantly reduced apparent fiber digestibility compared with normal diet (containing 16.70% TDF, control group). However, characteristics of microbiota at the species level and histological structure in pigs with the ability to digest appropriate high-fiber diets were still unknown. This study conducted comparative analysis of cecal physiology and microbial populations colonizing cecal mucosa. The results showed intestinal development indexes including cecum length, densities of cecal goblet cells, and renewal of cecal epithelial cells in treatment II and IV had better performance than those in the control. Paludibacter jiangxiensis, Coprobacter fastidiosus, Bacteroides coprocola CAG:162, Bacteroides barnesiae, and Parabacteroides merdae enriched in treatment II expressed large number of glycoside hydrolase (GH)-encoding genes and had the largest number of GH families. In addition, pathogenic bacteria (Shigella sonnei, Mannheimia haemolytica, and Helicobacter felis) were enriched in treatment IV. Correlation analysis revealed that the intestinal development index positively correlated with the relative abundance of cecal mucosal microbiota and the amount of digested fiber. These results indicated that increased proportions of fiber-degrading microbes and enhanced intestinal development jointly promote the host to digest an appropriate high-fiber diet. However, although too-high fiber levels in diet could maintain the adaptive development of cecal epithelium, the proportions of pathogenic bacteria increased, which might lead to a decrease of fiber digestion in pigs.

**IMPORTANCE** Although studies about the effects of dietary fiber on fiber digestion and intestinal microbiota of pigs were widely in progress, few studies have been conducted on the dynamic response of intestinal microbiota to dietary fiber levels, and the characteristics of intestinal microbiota and intestinal epithelial development adapted to high-fiber diet s were still unclear. Appropriate high fiber promoted the thickness of large intestine wall, increased the density of cecal goblet cells, and promoted the renewal of cecal epithelial cells. In addition, appropriate high fiber improves the microbial abundance with fiber-digesting potential. However, excessive dietary fiber caused an increase in the abundance of pathogenic bacteria. These results indicated that an increased proportion of fiber-degrading microbes and enhanced intestinal development jointly promote host to digest appropriate high-fiber diets. However, although too-high fiber levels in diet could maintain the adaptive development of cecal epithelium, the proportions of pathogenic bacteria increased, which might lead to a decrease of fiber digestion in pigs. Our data provided a theoretical basis for rational and efficient utilization of unconventional feed resources in pig production.

## INTRODUCTION

Trillions of microorganisms colonizing in the large intestines of mammals are closely related to host nutrient absorption and intestinal development (1–3). Dietary fiber, as the main reaction substrate of large intestine microbiota (4, 5), shapes the structure and modulates the functions of intestinal microbiota. Currently, a lot of fiber-rich ingredients were widely used in the diets for livestock, including pigs. However, various studies found that diets with a high fiber content could restrain the nutrient digestibility and feed intake in pigs (6, 7). Studies have shown that some Chinese local pig breeds have stronger tolerance to high-fiber diets than foreign commercial pigs (8, 9), and this characteristic of Chinese local pigs may be related to their developed large intestine (10, 11) and abundant fiber-degrading related intestinal microbiota (12).

Suhuai pigs, a new lean-type pig breed, which inherit 25% Chinese indigenous Huai pig ancestry and 75% Large White ancestry, possess excellent tolerance to high-fibrous feedstuffs (13, 14). In our previous published study, Suhuai pigs were fed with diets containing 16.70% (control group), 17.75% (treatment I), 19.10% TDF (treatment II), 20.05% (treatment III), and 24.11% (treatment IV) total dietary fiber (TDF) for 4 weeks (15), and the results showed that the apparent digestibility of insoluble dietary fiber (IDF) in treatment II had no significant difference with the control group but decreased in treatment IV. Then 16S rRNA sequencing was used to study the characteristics of microbial composition on cecal and colonic mucosa of Suhuai pigs in the control group, treatment II, and treatment IV and found that the microbial diversity of cecal mucosa increased, and three members of family *Lachnospiraceae* and two members of family *Ruminococcaceae* had higher relative abundance in treatment II, compared to those in the control group, whereas these adaptive changes disappeared in treatment IV. In addition, no effect of dietary fiber level was found on colonic mucosal microbial diversity, as well as abundance. Although the identification results of 16S rRNA gene sequencing at the genus level and at higher taxonomic levels are reliable, the sequencing resolution ratio at the species level is insufficient, resulting in the inability of functional analysis of microbiota. Shotgun metagenomic sequencing sequences the genomes of all the microbes isolated from the entire microbial community (16). Its advantage lies in the capacity for species-level reconstruction in the taxonomic analysis and for the functional annotation with pathway predictions of the studied microbiome (17). Barton et al. founded that shotgun metagenomic sequencing had greater fidelity and superior resolution of low levels of taxonomy (eg, species) than 16S rRNA gene sequencing (18). Therefore, shotgun metagenomic sequencing may help us to identify important microbes related to fiber digestion at species level.

Some studies using pigs as experimental animals found that high dietary fiber could facilitate the intestinal development of pigs. Kass et al. reported that feeding alfalfa powder to pigs would increase colon weight (19). Stanogias and Pearce found that dietary fiber affected the weight gain of all intestinal segments of pigs (20). Jørgensen et al. added fiber (268 g/kg) to the diet of finishing pigs and found that the length of large intestine, the weights of the cecum, colon, and total intestine of pigs increased (21). In addition, Fevrier et al. found that higher relative intestinal weight and length promoted the digestion of high-fiber diets in pigs (10). The above studies showed that there was a positive relationship between dietary fiber and intestinal development of pigs. However, few studies investigate the effects of dietary fiber on the proliferation and apoptosis of large intestinal epithelial cells in pigs.

The short-chain fatty acids (SCFAs), the end products of microbial fermentation of dietary fiber, are absorbed by intestinal epithelium (22, 23). Among them, acetate and propionate are transported to various organs of the whole body from the peripheral circulation to play biological functions (24). Butyrate is directly absorbed and used by intestinal epithelial cells. Roediger proved that the fatty acids of anaerobic bacteria are a major source of energy for the colonic mucosa using butyrate as fuels for epithelial cells (25). An *in vitro* study with normal human cecal biopsy specimens showed that SCFAs stimulated cell proliferation of the crypt cell (26). Goblet cells, producer of gastrointestinal mucin MUC2 (27, 28), are one of the main types of epithelial cells in the large intestine (29). Our previous study found that the proportions of cecal fiber-degrading bacteria and the yield of cecal SCFAs in pigs increased with an increase of dietary fiber level (15), and the mRNA expression of *MUC2* upregulated in large intestine (30). However, the previous study did not systematically investigate the structural characteristics of large intestine adapting to high fiber digestion. Lin et al. found that SCFAs promoted the expression of genes related to the development of rumen epithelial cells in sheep and provided a more suitable living environment for rumen microbiota, which enhanced the carbohydrate fermentation ability of gut microbiota in turn (31). This research indicates that there are interactions between intestinal microbiota and animal intestinal epithelial cells, which jointly promotes the nutrient digestion in ruminant. However, the characteristics of large intestinal microbiota and intestinal development of Chinese local or Suhuai pigs adapting to high-fiber diet are still unknown.

Consequently, this study hypothesized that the large intestinal microbiota and intestinal development of Suhuai pigs might jointly promote dietary fiber digestion. First, this study observed the dynamic characteristics of cecal epithelium of Suhuai pigs with an increase in the dietary fiber level. Subsequently, shotgun metagenomic sequencing was conducted to identify fiber-degrading cecal mucosal species and clarify their functional attributes. Finally, the relationships between cecal developmental indexes and cecal mucosal microbiota of pigs were comprehensively analyzed to reveal the potential roles of intestinal microbiota and intestinal development in the process of dietary fiber digestion.

## RESULTS

### Intestinal development and its relationships with digested fiber and SCFAs.

**FIG 1:**
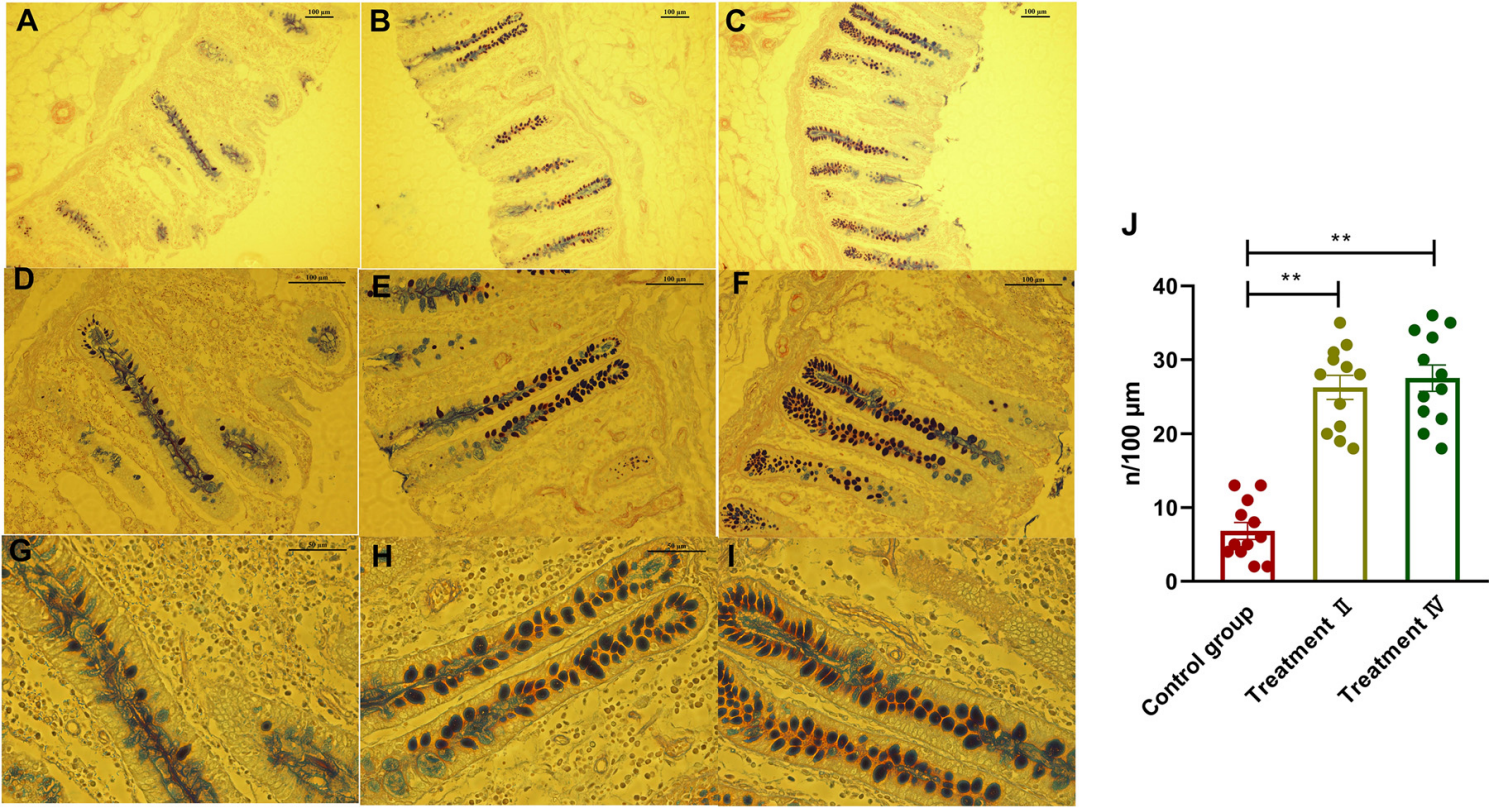
Alcian Blue/periodic acid-Schiff (AB-PAS) staining of goblet cells on cecal mucosa. (A, D, G) In the control group at ×100 magnification (A), ×200 magnification (D), and ×400 magnification (G). (B, E, H) With treatment II at ×100 magnification (B), ×200 magnification (E), and ×400 magnification. (C, F, I) With treatment IV at ×100 magnification (C), ×200 magnification (F), and ×400 magnification (I). Quantification of goblet cells numbers (J).

**FIG 2:**
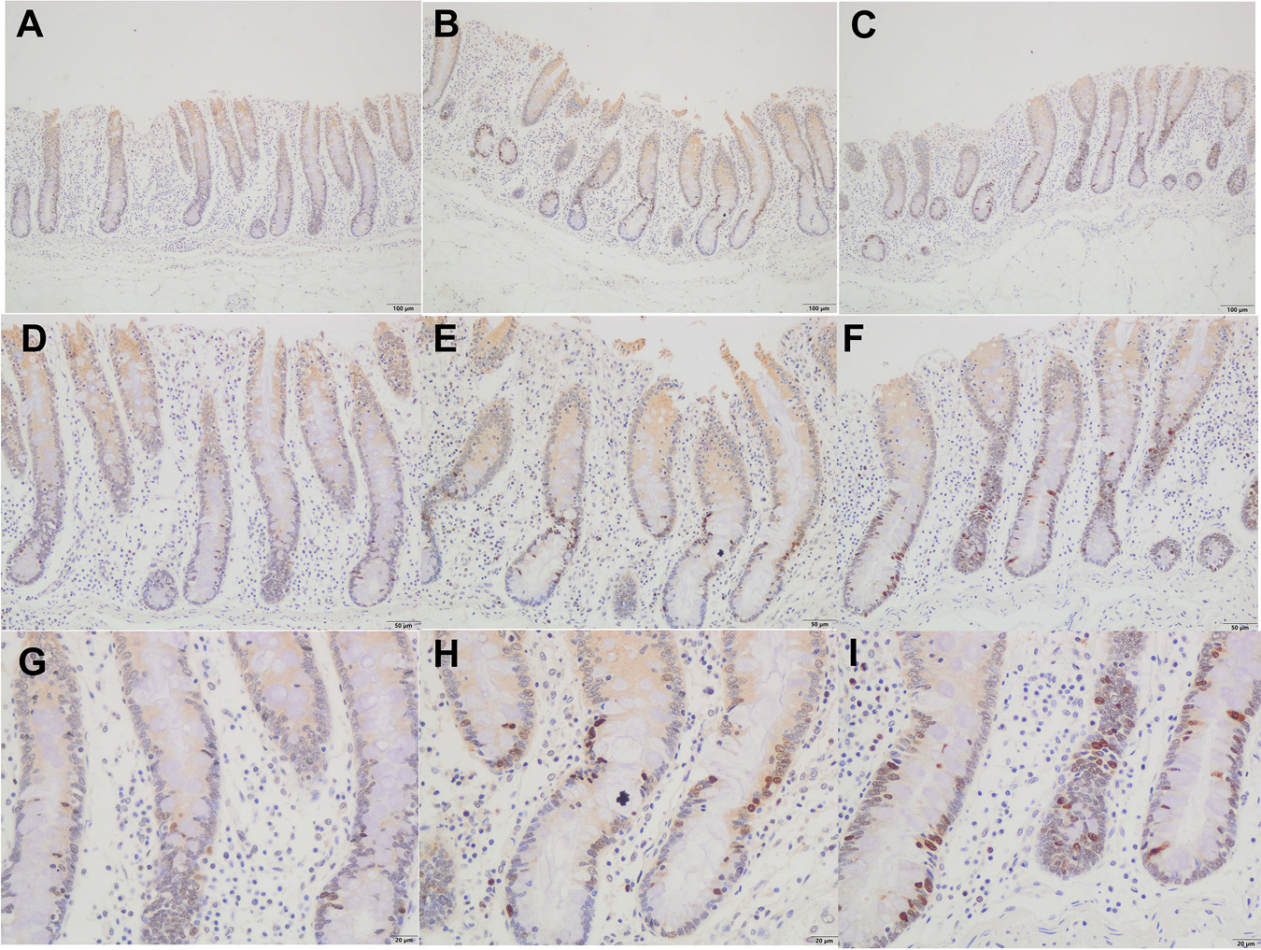
Ki-67 staining of cecal mucosa. (A, D, G) In the control group at ×100 magnification (A), ×200 magnification (D), and ×400 magnification (G). (B, E, H) With treatment II at ×100 magnification (B), ×200 magnification (E), and ×400 magnification (H). (C, F, I), With treatment IV at ×100 magnification (C), ×200 magnification (F), and ×400 magnification (I).

**FIG 3:**
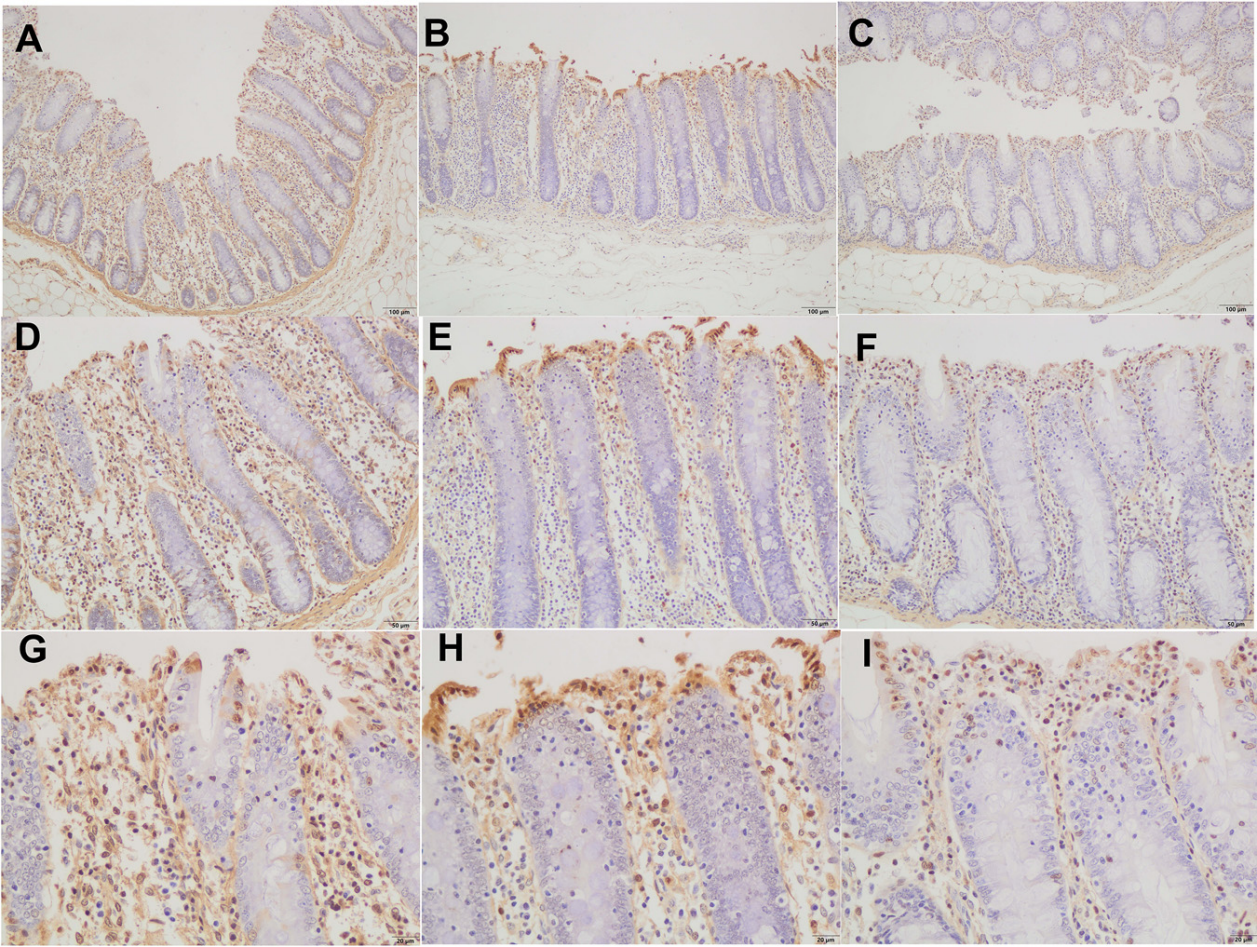
Terminal deoxynucleotidyltransferase-mediated dUTP-biotin nick end labeling (TUNEL) staining of cecal mucosa. (A, D, G) In the control group at ×100 magnification (A), ×200 magnification (D), and ×400 magnification (G). (B, E, H) With treatment II at ×100 magnification (B), ×200 magnification (E), and ×400 magnification (H). (C, F, I) With treatment IV ×100 magnification (C), ×200 magnification (F), and ×400 magnification (I).

With an increase of dietary fiber, cecum length increased (linear, *P < *0.05), and large intestine length tended to increase (linear, *P < *0.10) (Table 1). The other gastrointestinal morphometrics were not affected by dietary fiber level. The results of cecal mucosal morphology revealed that the submucosa thickness and intestinal wall thickness of treatments II and IV were higher than those of the control group (*P < *0.01) (Table 2), and the cecal mucosal morphology between treatment II and IV had no difference (Table 3). We further observed the histomorphology of cecal epithelium. Alcian Blue/periodic acid-Schiff (AB-PAS) staining revealed that the densities of goblet cells of cecum in both treatments II and IV were significantly higher than those in the control group (*P < *0.01) (Fig. 1). Similarly, the histomorphology of cecal epithelium between treatments II and IV had no difference (Fig. 1). The cell proliferation rates (Ki-67-positive cell percentage per crypt) of both treatments II and IV were significantly higher than those in the control group (*P < *0.01), and the cell proliferation rates of treatment IV were higher than those in treatment II (*P < *0.01) (Fig. 2; Table 3). In addition, the cell apoptosis rates of both treatments II and IV were significantly lower than those in the control group (*P < *0.01) (Fig. 3; Table 3). Interestingly, correlation analysis showed that densities of cecal goblet cells were positively correlated with the concentration of butyrate and total SCFAs (*P < *0.10) (Table S1), and the submucosa thickness, intestinal wall thickness, and goblet cells of cecum were positively correlated with the amount of digested IDF, soluble dietary fiber (SDF), and TDF (*P < *0.05) (Table S2).

**TABLE 1:** Effects of dietary fiber level on gastrointestinal morphometrics^*a*^

Absolute metrics	Groups			SEM	*P* value	
	Control	Treatment II	Treatment IV		ANOVA	Linear
Stomach weight (kg)	0.90	0.82	0.76	0.06	0.182	0.083
Cecum length (cm)	20.57	23.29	22.71	0.44	0.094	0.026
Small intestine weight (kg)	2.38	2.15	2.24	0.08	0.753	0.430
Large intestine weight (kg)	2.78	3.03	3.22	0.08	0.261	0.235
Small intestine length (cm)	1,882.5	2,092.71	1,964.43	34.71	0.452	0.441
Large intestine length (cm)	483.43	550.00	526.21	9.42	0.143	0.083

a An adjustment for multiple comparisons was conducted using least-significant difference (LSD). ANOVA, analysis of variance; SEM, standard error of the mean.

**TABLE 2:** Effects of dietary fiber level on cecal mucosal morphology^*a*^

Metrics	Group			SEM	*P* value	
	Control	Treatment II	Treatment IV		ANOVA	Linear
Crypt depth (μm)	452.03	462.07	448.66	6.66	0.715	0.844
Mucous thickness (μm)	542.1	484.79	491.68	15.36	0.259	0.186
Submucosa thickness (μm)	644.49^B^	1,116.35^A^	1,029.56^A^	59.26	0.001	0.002
Muscularis thickness (μm)	198.88	264.98	252.34	13.74	0.111	0.107
Intestinal wall thickness (μm)	1,385.47^B^	1,866.12^A^	1,773.57^A^	62.68	0.001	0.004

a In the same row, values with no letter or the same letter superscripts indicate no significant difference (*P *> 0.05), while values with different small letter superscripts indicate significant difference (*P *< 0.05), and values with different capital superscripts indicate significant difference (*P *< 0.01). ANOVA, analysis of variance; SEM, standard error of the mean.

**TABLE 3:** Effects of dietary fiber level on cell proliferation and apoptosis in cecal mucosa^*a*^

Items	Group			SEM	*P* value	
	Control	Treatment II	Treatment IV		ANOVA	Linear
Ki-67-positive cell percentage per crypt (%)	4.85^C^	21.67^B^	27.57^A^	1.88	<0.000	<0.000
TUNEL-positive cell percentage per 200 cecal mucosal cells (%)	30.75^A^	17.50^B^	19.00^B^	1.58	<0.000	<0.000

a In the same row, values with no letter or the same letter superscripts indicate no significant difference (*P *> 0.05), while values with different small letter superscripts indicate significant difference (*P *< 0.05), and values with different capital superscripts indicate significant difference (*P *< 0.01). ANOVA, analysis of variance; SEM, standard error of the mean; TUNEL, terminal deoxynucleotidyl transferase-mediated dUTP nick end labeling.

10.1128/msystems.00937-22.4 TABLE S1 Correlation analysis between short-chain fatty acids (SCFAs) and cecal mucosal morphology. Download Table S1, DOCX file, 0.01 MB. Copyright © 2022 Pu et al. 2022 Pu et al. https://creativecommons.org/licenses/by/4.0/ This content is distributed under the terms of the Creative Commons Attribution 4.0 International license.

10.1128/msystems.00937-22.5 TABLE S2 Correlation analysis between the amount of digested dietary fiber and cecal mucosal morphology. Download Table S2, DOCX file, 0.01 MB. Copyright © 2022 Pu et al. 2022 Pu et al. https://creativecommons.org/licenses/by/4.0/ This content is distributed under the terms of the Creative Commons Attribution 4.0 International license.

### Taxonomic configurations of cecal mucosal bacteria.

**FIG 4:**
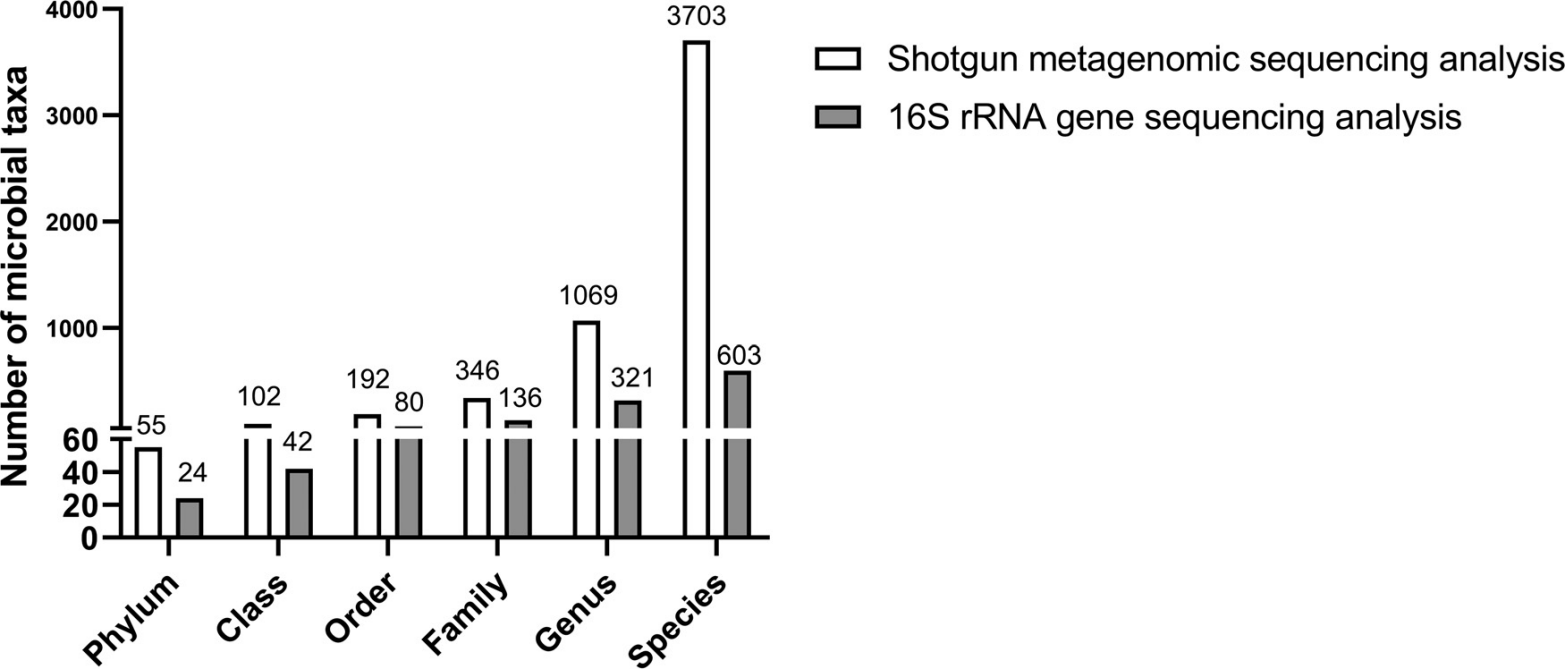
Numbers of microbial taxa by shotgun metagenomic sequencing and 16S rRNA gene sequencing.

After shotgun metagenomic sequencing of the 21 samples, a total of 247.33 Gb of raw data were obtained, which contained an average of 11.78 Gb (9.18 to 14.87 Gb) per sample. The sequence assembly analysis of 21 samples produced a total of 3.61 million contigs with an average length of 805 bp and an average *N*_50_ length of 1,011 bp. A total of 2,117,363 open reading frames (ORFs) were found with an average length of 221 bp (Table S3). Shotgun metagenomic sequencing analysis identified a larger microbial population than our previous findings by 16S rRNA gene sequencing (SRP155), which provided us with abundant microbial information at species level to identify the species associated with high fiber digestion of Suhuai pigs (Fig. 4; Table S4). Microbial composition analysis showed that *Bacteroidetes* was the dominant phylum (69.46%) in the cecum mucosa, followed by the phylum *Firmicutes* (16.64%) and the phylum *Proteobacteria* (8.06%). At the genus level, Prevotella and Bacteroides, as the members of phylum *Bacteroidetes*, were the dominant genera (48.52%, 12.51%) in the cecum mucosa, followed by Campylobacter (2.82%) (Fig. S1).

10.1128/msystems.00937-22.1 FIG S1 Information of KEGG annotation analysis of different gene set (DGS). Download FIG S1, TIF file, 1.3 MB. Copyright © 2022 Pu et al. 2022 Pu et al. https://creativecommons.org/licenses/by/4.0/ This content is distributed under the terms of the Creative Commons Attribution 4.0 International license.

10.1128/msystems.00937-22.6 TABLE S3 The sequence assembly analysis of shotgun metagenomic sequencing. Download Table S3, DOCX file, 0.02 MB. Copyright © 2022 Pu et al. 2022 Pu et al. https://creativecommons.org/licenses/by/4.0/ This content is distributed under the terms of the Creative Commons Attribution 4.0 International license.

10.1128/msystems.00937-22.7 TABLE S4 Information of microbial taxa by different sequencing methods. Download Table S4, DOCX file, 0.01 MB. Copyright © 2022 Pu et al. 2022 Pu et al. https://creativecommons.org/licenses/by/4.0/ This content is distributed under the terms of the Creative Commons Attribution 4.0 International license.

**FIG 5:**
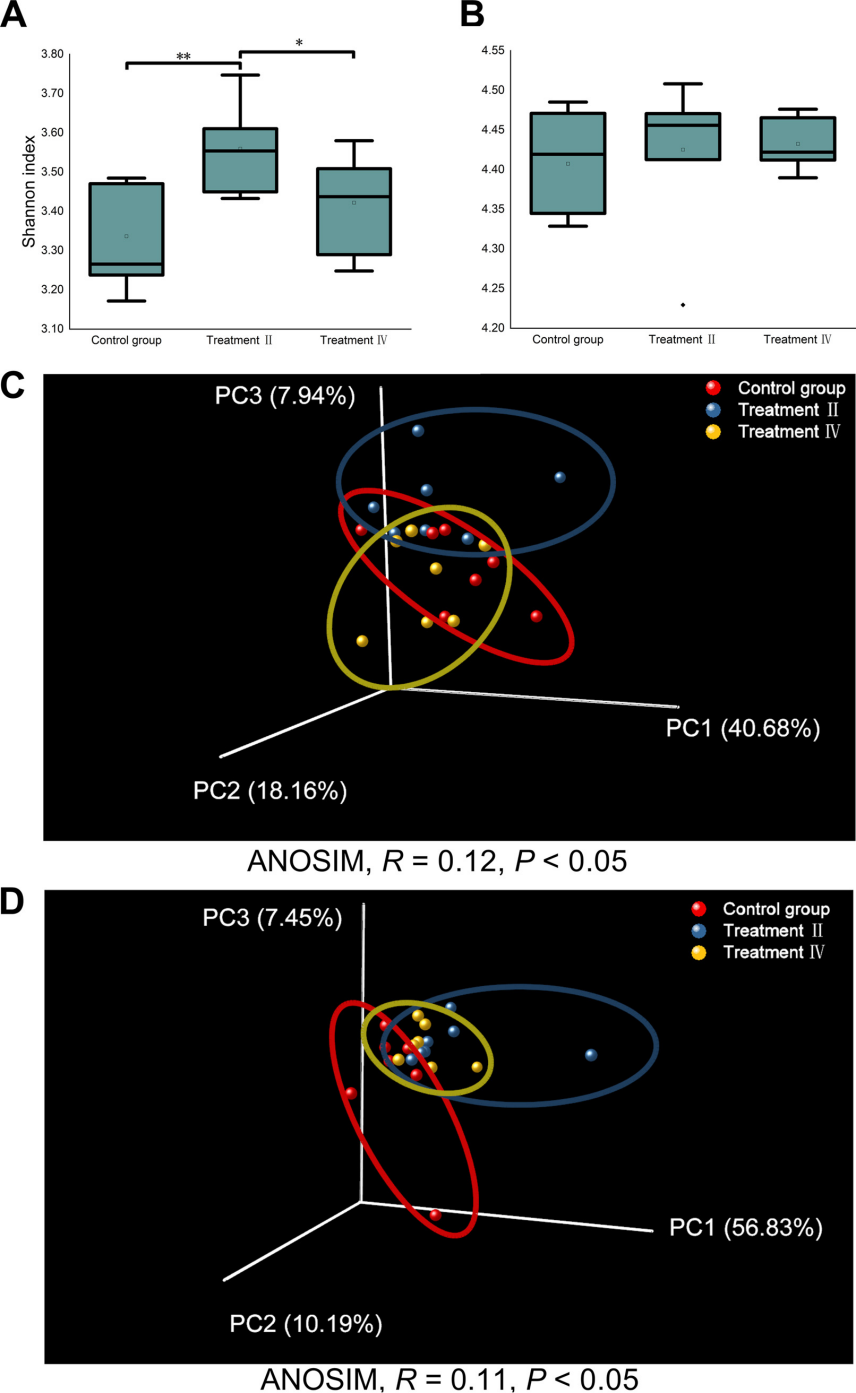
Microbial diversity of cecal mucosa. (A, B) The microbial α diversity (Shannon index) at species level (A) and carbohydrate-active enzymes (CAZyme) function level (B). (C, D) Principal coordinate analysis (PCoA) analysis based on Bray_Curtis distance at species level (C) and CAZyme function level (D). ANOSIM, analysis of similarities.

The microbial α diversity (Shannon index) at species level in treatment II were higher than those in the other two groups (*P* < 0.05) (Fig. 5A), while there was no significant difference in the Shannon index of the carbohydrate-active enzymes (CAZymes) among three groups. In addition, the Shannon index had no significant difference between treatment IV and the control group. Meanwhile, Principal coordinate analysis (PCoA) analysis revealed that both microbial composition at species level (analysis of similarities [ANOSIM], *R *= 0.12, *P < *0.05) and CAZyme functions (ANOSIM, *R *= 0.11, *P < *0.05) in treatment II differed from those in the control group, while the microbial compositions at species level and in CAZyme functions of treatment IV were close to treatment II and the control group (Fig. 5C and D).

Wilcoxon rank-sum test showed that 70 species with significant differences between treatment II and the control group were detected (*P < *0.01). Among them, there were 66 species in treatment II that had higher relative abundance than those in the control group (Table 4), and these 66 species showed no differences between treatments II and IV (Table S5).

**TABLE 4:** The 66 bacteria at species level enriched in treatment II^*a*^

Items	Control group		Treatment II		*P* value
	Mean (%)	SD	Mean (%)	SD	
Coprobacter secundus	0.0605	0.0375	0.2274	0.0611	0.0022
Bacteroides barnesiae	0.1221	0.0295	0.2285	0.0485	0.0073
Coprobacter fastidiosus	0.0365	0.0239	0.1212	0.0388	0.0049
Unclassified f *Porphyromonadaceae*	0.0264	0.0275	0.1099	0.0284	0.0022
Intestinimonas butyriciproducens	0.0288	0.0203	0.0965	0.0497	0.0073
Peptoclostridium difficile	0.0514	0.0153	0.1117	0.0340	0.0022
Bacteroides sp. CAG:770	0.0732	0.0200	0.1324	0.0469	0.0073
Parabacteroides merdae	0.0756	0.0257	0.1316	0.0273	0.0049
Tannerella sp. CAG:51	0.0145	0.0094	0.0704	0.0189	0.0022
Paludibacter jiangxiensis	0.0356	0.0121	0.0912	0.0454	0.0073
Eubacterium plexicaudatum	0.0863	0.0299	0.1371	0.0240	0.0073
Oscillibacter sp. KLE 1745	0.0147	0.0106	0.0585	0.0356	0.0073
Bacteroides sp. CAG:20	0.0205	0.0129	0.0632	0.0191	0.0049
Dysgonomonas mossii	0.0258	0.0134	0.0633	0.0171	0.0049
Dysgonomonas capnocytophagoides	0.0205	0.0146	0.0531	0.0086	0.0033
Uncultured bacterium fosmid pJB154B8 contig II	0.0103	0.0086	0.0401	0.0132	0.0073
Dysgonomonas sp. HGC4	0.0114	0.0099	0.0410	0.0139	0.0049
Fibrobacter succinogenes	0.0236	0.0099	0.0523	0.0098	0.0033
Dysgonomonas sp. BGC7	0.0092	0.0081	0.0367	0.0125	0.0022
Dorea formicigenerans	0.0192	0.0097	0.0464	0.0187	0.0049
Odoribacter sp. CAG:788	0.0146	0.0060	0.0411	0.0168	0.0033
Bacteroidales bacterium Barb6XT	0.0149	0.0097	0.0381	0.0087	0.0022
*Ruminococcaceae* bacterium cv2	0.0154	0.0089	0.0369	0.0120	0.0033
Bacteroides propionicifaciens	0.0148	0.0067	0.0362	0.0135	0.0049
Alistipes sp. CAG:268	0.0065	0.0042	0.0265	0.0224	0.0073
Alistipes finegoldii	0.0162	0.0047	0.0356	0.0079	0.0022
[Eubacterium] hallii	0.0119	0.0068	0.0306	0.0142	0.0073
Veillonella montpellierensis	0.0149	0.0077	0.0333	0.0089	0.0049
Treponema denticola	0.0094	0.0058	0.0278	0.0148	0.0073
Uncultured bacterium fosmid pJB89E1	0.0129	0.0052	0.0313	0.0119	0.0033
Butyrivibrio sp. AE3003	0.0101	0.0075	0.0284	0.0067	0.0049
Bacteroides coprocola CAG:162	0.0217	0.0055	0.0393	0.0107	0.0073
Porphyromonas gingivicanis	0.0098	0.0077	0.0249	0.0079	0.0073
Porphyromonas asaccharolytica	0.0126	0.0047	0.0266	0.0082	0.0033
Sanguibacteroides justesenii	0.0076	0.0065	0.0214	0.0056	0.0049
Uncultured bacterium Contigcl 1748	0.0069	0.0060	0.0206	0.0052	0.0049
Bacteroidales bacterium KA00251	0.0047	0.0039	0.0181	0.0042	0.0033
Uncultured rumen bacterium	0.0017	0.0026	0.0151	0.0066	0.0033
Uncultured bacterium fosmid pJB154B8 contig I	0.0015	0.0016	0.0134	0.0049	0.0021
Uncultured bacterium fosmid pJB102C1	0.0016	0.0025	0.0126	0.0068	0.0032
[Eubacterium] siraeum	0.0060	0.0045	0.0169	0.0063	0.0073
Firmicutes bacterium CAG:83	0.0027	0.0024	0.0132	0.0109	0.0049
Porphyromonas levii	0.0051	0.0034	0.0156	0.0048	0.0049
Proteiniphilum sp. 51 7	0.0021	0.0021	0.0121	0.0073	0.0072
Clostridium sp. ATCC BAA-442	0.0109	0.0032	0.0206	0.0063	0.0022
Clostridium nexile CAG:348	0.0016	0.0024	0.0105	0.0060	0.0048
Uncultured bacterium URE4	0.0013	0.0016	0.0092	0.0073	0.0072
Clostridium sp. CAG:264	0.0061	0.0031	0.0139	0.0046	0.0049
Porphyromonas sp. COT-290 OH860	0.0019	0.0026	0.0095	0.0045	0.0048
Porphyromonas crevioricanis	0.0052	0.0029	0.0128	0.0051	0.0033
Solitalea canadensis	0.0013	0.0019	0.0086	0.0044	0.0067
Eggerthella sp. CAG:1427	0.0054	0.0040	0.0127	0.0037	0.0073
Ruminococcus sp. CAG:379	0.0028	0.0029	0.0095	0.0045	0.0073
Alistipes finegoldii CAG:68	0.0010	0.0013	0.0066	0.0047	0.0070
Hymenobacter norwichensis	0.0018	0.0019	0.0071	0.0032	0.0070
Youngiibacter fragilis	0.0032	0.0025	0.0080	0.0025	0.0073
Cellulophaga lytica	0.0008	0.0009	0.0050	0.0049	0.0072
Empedobacter brevis	0.0006	0.0007	0.0042	0.0032	0.0070
Firmicutes bacterium CAG:345	0.0005	0.0009	0.0040	0.0010	0.0019
Phaeodactylibacter xiamenensis	0.0001	0.0003	0.0034	0.0022	0.0022
Chryseobacterium palustre	0.0000	0.0000	0.0033	0.0028	0.0037
Algoriphagus terrigena	0.0003	0.0006	0.0036	0.0024	0.0040
Mesoflavibacter zeaxanthinifaciens	0.0008	0.0009	0.0037	0.0017	0.0048
Denitrobacterium detoxificans	0.0005	0.0008	0.0033	0.0020	0.0045
Firmicutes bacterium CAG:449	0.0002	0.0005	0.0026	0.0020	0.0064
Polaribacter sp. Hel I 88	0.0000	0.0000	0.0019	0.0016	0.0037
Lacinutrix sp. 5H-3-7-4	0.0037	0.0047	0.0000	0.0001	0.0064
Helicobacter sp. MIT 09-6949	0.0151	0.0120	0.0030	0.0034	0.0073
Bacteroides sp. D20	0.0618	0.0614	0.0254	0.0065	0.0073
Megasphaera elsdenii	0.1219	0.0972	0.0316	0.0168	0.0073

a The difference between treatment II and the control group was tested by Wilcoxon rank-sum test.

10.1128/msystems.00937-22.8 TABLE S5 The 66 species with no differences between treatments II and IV. Download Table S5, DOCX file, 0.02 MB. Copyright © 2022 Pu et al. 2022 Pu et al. https://creativecommons.org/licenses/by/4.0/ This content is distributed under the terms of the Creative Commons Attribution 4.0 International license.

Then, the species in treatment IV that had significant different relative abundance with other groups were also detected. The results showed that there were 20 species with significant differences between treatment IV and the control group (*P < *0.01) (Table S6) and 49 species with significant differences between treatment IV and treatment II (*P < *0.01) (Table S7). It is worth noting that the well known pathogenic bacteria Shigella sonnei (32), Mannheimia haemolytica (33), and Helicobacter felis (34) had higher relative abundance in treatment IV compared to the control group and treatment II.

10.1128/msystems.00937-22.9 TABLE S6 The 20 species with significant differences between treatment IV and the control group. Download Table S6, DOCX file, 0.01 MB. Copyright © 2022 Pu et al. 2022 Pu et al. https://creativecommons.org/licenses/by/4.0/ This content is distributed under the terms of the Creative Commons Attribution 4.0 International license.

10.1128/msystems.00937-22.10 TABLE S7 The 49 species with significant differences between treatments IV and II. Download Table S7, DOCX file, 0.02 MB. Copyright © 2022 Pu et al. 2022 Pu et al. https://creativecommons.org/licenses/by/4.0/ This content is distributed under the terms of the Creative Commons Attribution 4.0 International license.

### Fiber-degrading-related functions of the cecal mucosal microbiota.

**FIG 6:**
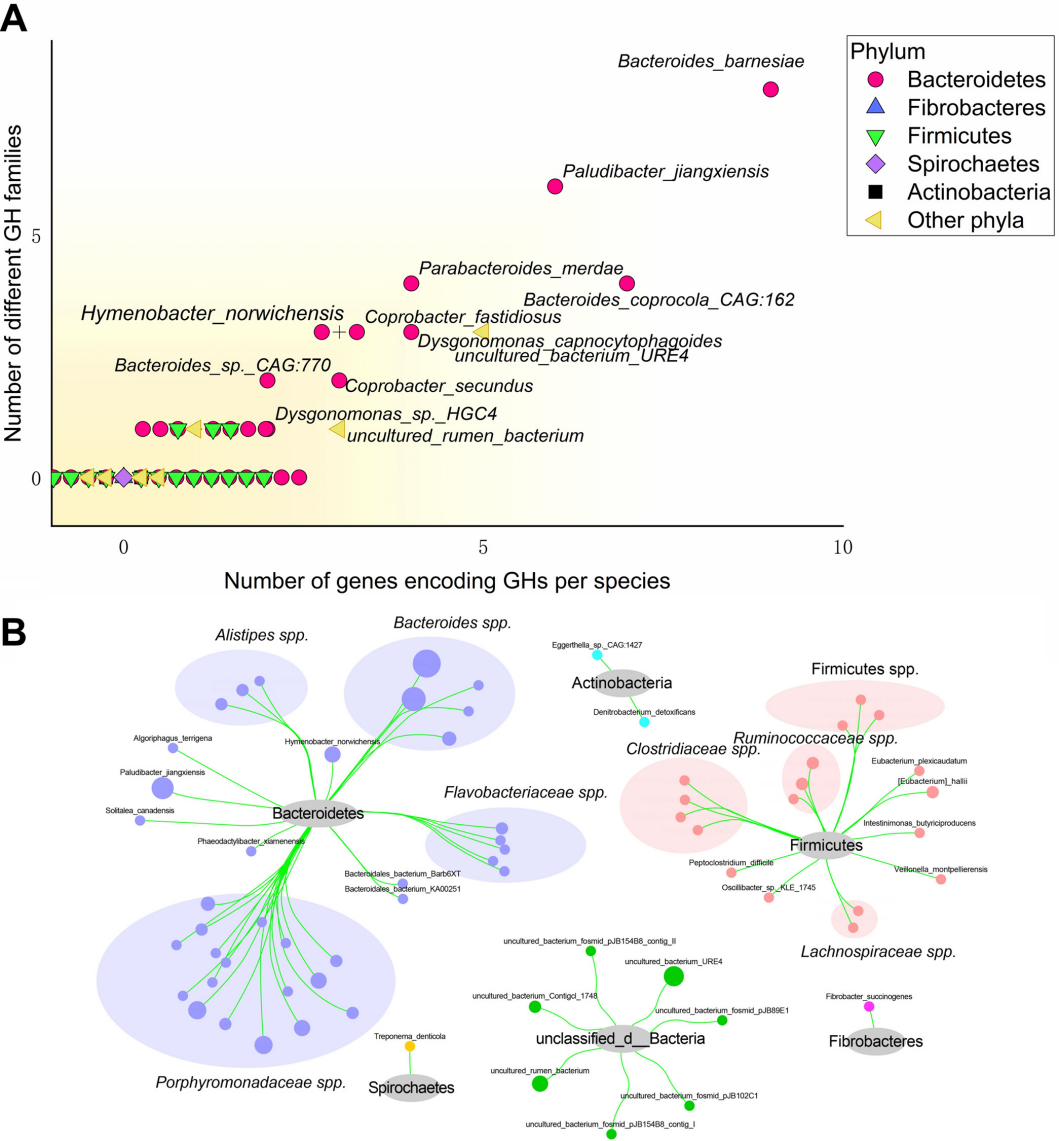
The expression of genes encoding glycoside hydrolase (GHs) in different gene sets (DGSs). (A) Numbers of genes encoding GHs in the genomes constituting the DGS, and the numbers of GH families represented in DGS. The label + means the points around here coincided. (B) Community distribution of 66 species containing genes encoding GHs. The size of target nodes represents the number of genes encoding GHs.

**FIG 7:**
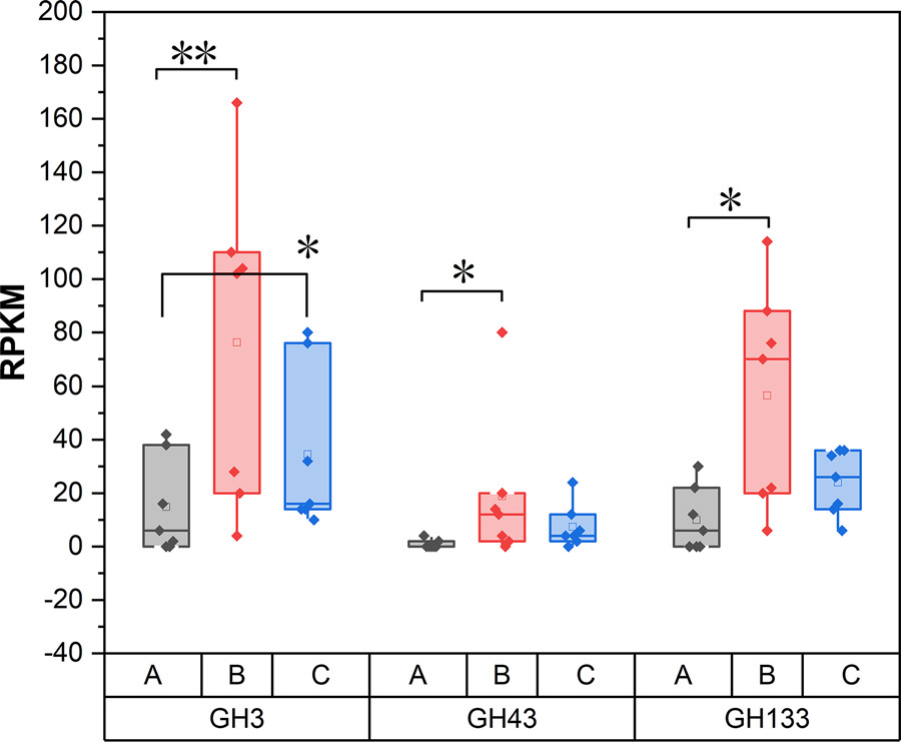
Relative abundances of GH3, GH43, and GH33 in each group. A indicates the control group; B indicates treatment II; and C indicates treatment IV. RPKM, reads per kilobase per million.

The functions of the 66 species significantly enriched in treatment II were further analyzed to explore the reason why pigs in treatment II could digest more fiber to maintain fiber apparent digestibility. The genes of 66 species enriched in treatment II were used to build different gene set (DGS) by Tolerance CD-HIT software (http://www.bioinformatics.org/cd-hit/) with the following parameters: 90% similarity and 90% coverage threshold. Then, 2,123 catalog genes in DGS were obtained, with an average length of 578.23 bp. CAZymes encoded by the intestinal microbiome could catalyze the breakdown of glycoconjugates, oligosaccharides, and polysaccharides to fermentable monosaccharides. To specifically explore the microbial potential for dietary fiber degradation in the DGS, we screened for CAZyme-encoding genes in DGS. The number of genes encoding glycoside hydrolases (GHs) in DGS plotted against the number of GH families to which they belong (Fig. 6). A total of 61 GH-encoding genes and 51 GH family numbers were annotated. Paludibacter jiangxiensis, Coprobacter fastidiosus, Bacteroides coprocola CAG:162, Bacteroides barnesiae, and Parabacteroides merdae expressed the largest number of GH-encoding genes and had the largest number of GH families in DGS (Fig. 6A). *Porphyromonadaceae* spp. And Bacteroides spp. Contained the most GH-encoding genes (72.13%) and also had the most members of GH families (72.55%) (Fig. 6). We further compared the GHs in the DGS of each of the two groups and found that the relative abundances of GH3, GH43, and GH33 in treatment II were significantly higher than those in the control group (*P < *0.05, Fig. 7).

**FIG 8:**
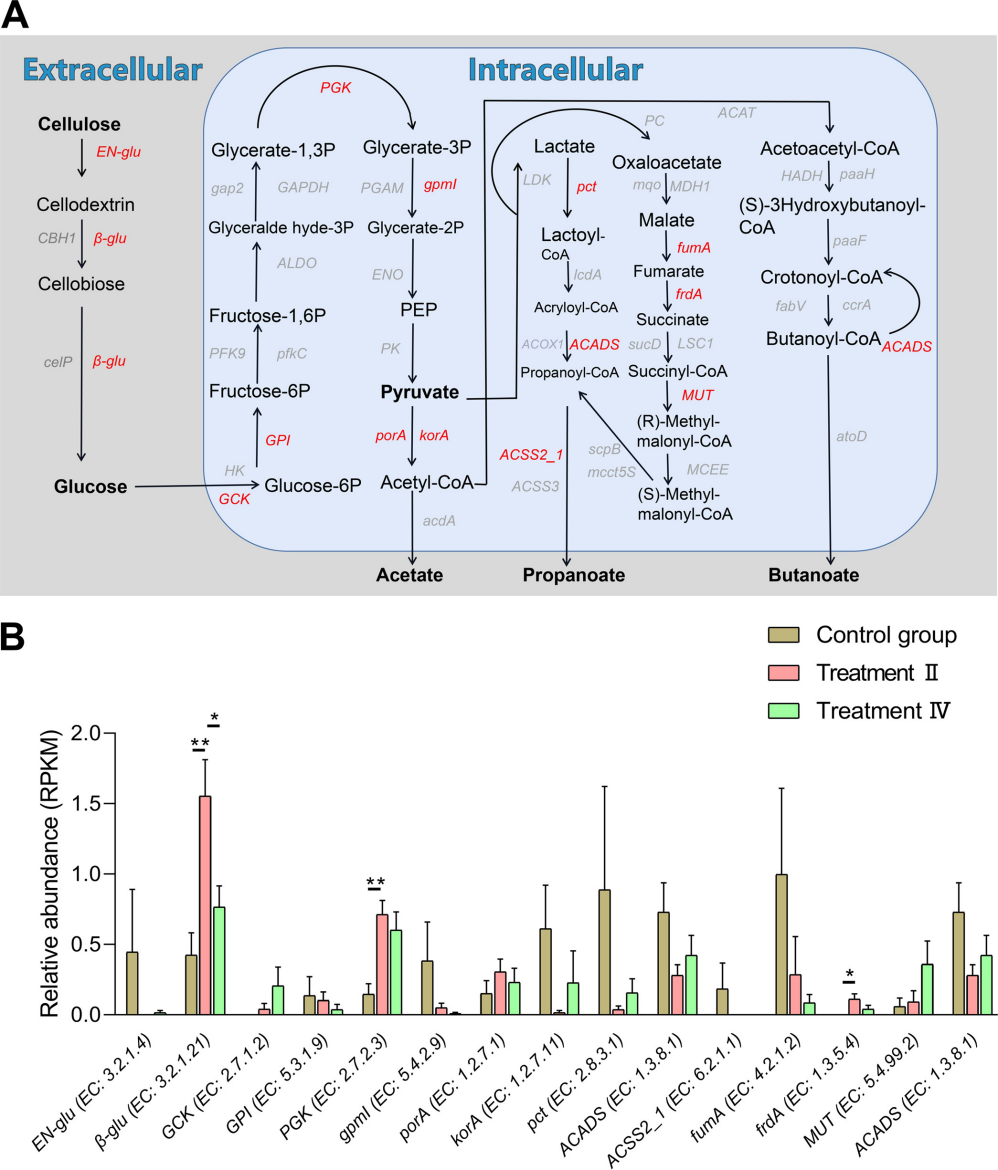
Process of dietary fiber metabolism. (A) The main functional pathways, intermediate metabolites of fiber metabolism and major genes. (B) Difference analysis of the 15 genes annotated by DGS; *, *P < *0.05. **, *P < *0.01.

In order to explore the microbial potential of fiber degradation and producing SCFAs, DGS was annotated to the KEGG database. KEGG annotation analysis of DGS revealed that a total of 150 KEGG pathways at level 3 were enriched (Fig. S2). In general, complex fiber structure converts to fermentable monosaccharides and will go through the following pathways in turn: starch and sucrose metabolism (ko00500), glycolysis/gluconeogenesis (ko00010), pyruvate metabolism (ko00620), butanoate metabolism (ko00650), and propanoate metabolism (ko00640) (35). To sort out the expression of genes coding enzymes involved in fiber degradation in these pathways, the major enzymes in these pathways were searched, and 15 enzymes were successfully annotated by using DGS (Fig. 8A). Among these 15 enzymes, *β-glu* (β-glucosidase), *PGK* (phosphoglycerate kinase), and *FRDA* (fumarate reductase flavoprotein subunit) had higher abundance in treatment II than those in the control group (Fig. 8B).

10.1128/msystems.00937-22.2 FIG S2 Microbial composition at the phylum (A) and the genus level (B). Phyla with relative abundance below 0.1% were classified as other, and genera with relative abundance below 1% were classified as other. Download FIG S2, TIF file, 1.6 MB. Copyright © 2022 Pu et al. 2022 Pu et al. https://creativecommons.org/licenses/by/4.0/ This content is distributed under the terms of the Creative Commons Attribution 4.0 International license.

### Relationships among relative abundance of cecal mucosal microbiota, development indexes of large intestine, and amount of digested dietary fiber.

**FIG 9:**
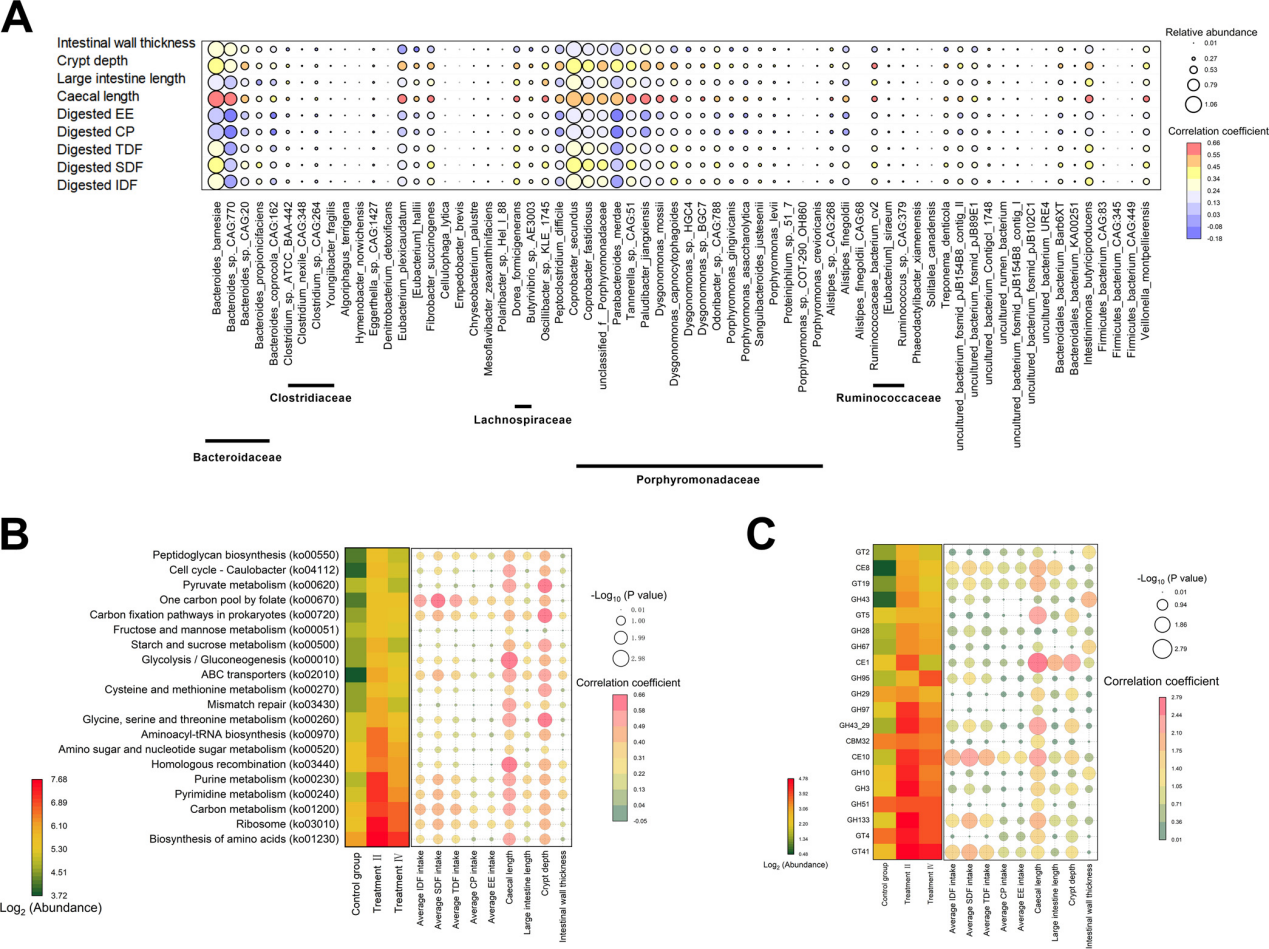
(A) Spearman’s correlation analysis (two-tailed test) between large intestine development indexes, amounts of digested crude protein (CP), ether extract (EE), insoluble dietary fiber (IDF), soluble dietary fiber (SDF), total dietary fiber (TDF) with the 66 species. (B) Main KEGG pathways (top 20). (C) Main CAZYme families.

To investigate the relationships among relative abundance of cecal mucosal microbiota, the development indexes of large intestines, and the amount of digested dietary fiber, Spearman’s correlation coefficient analysis was performed, and some compact relationships among them were detected. For instance, the amount of digested IDF, SDF, and TDF positively correlated with 3, 8, and 3 species, respectively (*P < *0.05) (Fig. 9A). Remarkably, cecal length and crypt depth positively correlated with 49 and 30 species, respectively (*P < *0.05) (Fig. 9A). Then, correlation analysis among the cecal development index, the amount of digested dietary compositions, and the relative abundance of microbial functions was further conducted. Similarly, cecal development indexes also had the most positive relationships with relative abundance of microbial functions. To be specific, cecal length positively correlated with 14 KEGG pathways (top 20 pathways annotated by DGS) and 12 CAZyme families (top 20 families annotated by DGS), respectively (*P < *0.05) (Fig. 9B and C). In addition, the amount of digested IDF, SDF, and TDF positively correlated with 3, 5, and 3 KEGG pathways and 3, 4, and 3 CAZyme families, respectively (*P < *0.05; Fig. 9B and C). In particular, the expression profile of carbohydrate metabolism-related pathways and their relationship with environmental factors were further investigated. Similar to the above results, cecum length and crypt depth of cecum had generally positive correlations with carbohydrate metabolism-related pathways (Fig. S3).

10.1128/msystems.00937-22.3 FIG S3 Spearman’s correlation analysis (two-tailed test) between large intestine development indexes, amounts of digested crude protein (CP), ether extract (EE), insoluble dietary fiber (IDF), soluble dietary fiber (SDF), and total dietary fiber (TDF) with the 15 KEGG pathways in carbohydrate metabolism (pathway level 2). Download FIG S3, TIF file, 1.0 MB. Copyright © 2022 Pu et al. 2022 Pu et al. https://creativecommons.org/licenses/by/4.0/ This content is distributed under the terms of the Creative Commons Attribution 4.0 International license.

## DISCUSSION

The large intestine of pigs is the main position where dietary fiber is digested and utilized (36). Some animal studies had shown that high intake of dietary fiber would affect the development of digestive tract of pigs, especially the large intestine (19, 37, 38). Kass et al. (19) found that the weight of the empty gastrointestinal tract (all segments except stomach) increased with the increase of dietary fiber level. Similarly, Van Hees et al. (39) used a dietary fiber-enriched diet to feed suckling piglets and found that dietary fiber enrichment in diet increased the relative weight of the large intestine. A previous study focused on the difference of fiber digestion in pigs but lacked any analysis of the intestinal structure characteristics of pigs adapting to high-fiber diets (15). To investigate the intestinal development characteristics of pigs fed with different fiber levels, microscopic observation of cecal epithelium was first conducted. Consistent with these previous studies, the increase of dietary fiber level promoted an increase of cecum length and large intestine length and the proliferation of cecal goblet cells and crypt cells in the present study. In addition, the thickness of cecal submucosa and total thickness increased with the increase of the dietary fiber level as well.

Dietary fiber is fermented mainly by intestinal microbiota in large intestine, and the main metabolites are SCFAs. SCFAs can be directly absorbed by the intestinal wall to provide energy for intestinal epithelial cells. In our previous study, the concentration of SCFAs in cecum increased with the dietary fiber level. Interestingly, the density of cecal goblet cells in this study was positively correlated with SCFAs in cecum, which is consistent with the findings by Piekarska et al. (40). Therefore, increasing the dietary fiber led to development of the large intestine in Suhuai pigs, which might be mainly caused by the more SCFAs production. However, it should be noted that when the level of TDF reached 24.11%, further development of the cecal epithelium did not happen, which suggested that intestinal development is not a simple linear relationship with dietary fiber level.

As the enzymes secreted by pigs cannot degrade dietary fiber, large microbiota harbored by large intestine are required to ferment and utilize fiber (35). Dietary fiber, as the main substrate of intestinal microbiota, plays a considerable role in microbial scale and functions (41, 42). In our previous study (15), pigs in treatment II had higher relative abundance of a constellation of fiber-degrading genera, particularly the unclassified f *Ruminococcaceae*, *Ruminococcaceae* UCG-010, no rank f *Lachnospiraceae* (43), Acetitomaculum (44), and Butyrivibrio (45), compared to the control group. In order to identify microbiota related to fiber degradation in cecal mucosa at the species level and clarify their functional characteristics, we conducted metagenomic sequencing in the present study. In particular, we identified 2 species in *Lachnospiraceae*, 3 species in *Ruminococcaceae*, 18 species in *Porphyromonadaceae*, and 5 species in *Bacteroidaceae* enriched in treatment II that were reported to be dominant fiber-degrading microbiota (43, 46).

Cellulose (main component of plant cell wall) is composed of a single glucose through β-(1,4)-glycosidic bonds (47). Possessing the enzymes that can breakdown these glycosidic bonds is necessary to utilize cellulose in plants. CAZymes encoded by the gut microbiome catalyze the breakdown of glycoconjugates, oligosaccharides, and polysaccharides to fermentable monosaccharides. Among them, GHs cleaved glycosidic bonds between carbohydrates by the insertion of a water molecule (hydrolysis) (48, 49). To investigate the expression of fiber-degrading related genes in DGS, DGS was annotated against the CAZyme database and KEGG pathway. The relative abundance of families (GH133, GH43, and GH3) and KEGG enzymes (*β-glu*, *PGK*, and *FRDA*) that had significant roles in the process of the fermentation of polysaccharides and oligosaccharides to SCFAs was significantly higher in treatment II than those in the control group. These results indicated that the microbes enriched in treatment II possessed the potential of fiber digestion.

The microbiota, especially *Bacteroidetes*, are believed to complement eukaryotic genomes with degradation enzymes targeting resistant dietary polymers, such as plant cell wall compounds (e.g., cellulose, pectin, and xylan) (46), and phylum *Bacteroidetes* possessed the highest potential of fiber degradation compared to other phyla (43, 50). In the present study, we noted that P. jiangxiensis, C. fastidiosus, B. coprocola CAG:162, B. barnesiae, and P. merdae (five members belonging to the phylum *Bacteroidetes*) expressed the largest number of GH-encoding genes and had the largest number of GH families in DGS. Research based on bacterial culture recorded that these five species were efficient SCFA producers and had polysaccharide-degrading functions (51–54). However, the colonization of the above five species on cecal mucosa in pigs and their responses to dietary fiber level have not been reported. Our study revealed that appropriate dietary fiber levels (TDF, 19.10%) promoted the colonization of above-mentioned fiber-degrading species on cecal mucosa, which provided reliable evidence for explaining the high fiber digestibility of Suhuai pigs. It was worth noting that a too-high level of dietary fiber (TDF, 24.11%) caused an increase in the relative abundance of three pathogenic microbes. Previous studies reported that S. sonnei, M. haemolytica, and H. felis were the leading causes of diarrhea (55), malnutrition (56), and even death of livestock (34). Therefore, the decrease of fiber apparent digestibility in pigs fed high-fiber diets (TDF, 24.11%) might be caused by the increased relative abundance of cecal mucosal pathogen.

Other livestock research had shown that SCFAs produced by fiber metabolism promoted the development of the intestine, which better promoted the propagation of functional microbiota and increased fiber digestion (31). Therefore, correlation analysis between intestinal structure and functional microbes in pigs was carried out in the present study. The results showed that cecal length and crypt depth positively correlated with 49 and 30 species, respectively. Concurrently, cecal length positively correlated with 14 KEGG pathways (top 20 pathways annotated by DGS) and 12 CAZyme families (top 20 families annotated by DGS), respectively. These results indicated that increased proportions of fiber-degrading microbes and enhanced intestinal development might jointly promote host to digest appropriate high-fiber diets in Suhuai pigs.

### Conclusion.

The intestinal development indexes (cecal morphology, densities of cecal goblet cells, and renewal of cecal epithelial cells) of Suhuai pigs were improved with a high-fiber diet (TDF, 19.10% and 24.11%). There were 66 species in pigs fed with diet containing 19.10% TDF that had higher relative abundance than those in the control group. Among them, P. jiangxiensis, C. fastidiosus, B. coprocola CAG:162, B. barnesiae, and P. merdae expressed the largest number of glycoside hydrolases (GHs)-encoding genes and have the largest number of GH families. In addition, the pathogenic bacteria (S. sonnei, M. haemolytica, and H. felis) were enriched in pigs fed with a diet too high in fiber (TDF, 24.11%). Correlation analysis revealed cecal length and crypt depth positively correlated with abundance of 49 and 30 species, respectively. Concurrently, cecal length positively correlated with abundance of 14 KEGG pathways (top 20 pathways annotated by DGS) and 12 CAZyme families (top 20 families annotated by DGS), respectively. In addition, part of intestinal development indexes (submucosa thickness, intestinal wall thickness, and densities of cecal goblet cells) positively correlated with the amount of digested IDF, SDF, and TDF. These results indicated that increased proportions of fiber-degrading microbes and enhanced intestinal development jointly promote host to digest appropriate high-fiber diets (TDF, 19.10%). However, although too high a fiber level in the diet (TDF, 24.11%) could maintain the adaptive development of cecal epithelium, the proportions of pathogenic bacteria increased, which might lead to the decreasing of fiber digestibility in pigs.

## MATERIALS AND METHODS

### Experimental design, diets, and management.

The experimental design and sample for the present study were derived from our previous study. The details of experimental design, selection of experimental animals, animal feeding, and management were described in detail in the previous study (15). Briefly, a total of 35 Suhuai barrows with body weights of 62.90 ± 0.78 kg were selected and allotted into 5 groups: the control group and treatments I to IV using a completely randomized design. All pigs were fed by the Osborne testing stations system (OTSS, provided by OSB Livestock Technology Co., Ltd., Shanghai, China). In the OTSS, there is a testing station for each pen. The testing station mainly consists of a weight scale, a feed scale, a feeding trough, an ear tag identification system, and a data transmission system. When the pigs enter the testing station and start feeding, the identification system in the feeding trough can quickly identify the pigs’ electronic ear tags. At the same time, the scale and feed scale will detect and record the pig’s weight and feed intake. By adjusting the width of the station, we ensure that only one pig can enter the station at a time. Each day, we can get the weight data and feed intake data of each pig from the previous day. The OTSS can accurately record daily intake and body weight individually; thus, each pig is identified as a replicate, and there are 7 replicates in each group. During the prefeeding period of 10 days, all pigs were fed with the basal diet. During the 28 days of the trial period, the pigs in the control group and treatment groups I to IV were provided with different diets: the basal diet (the same basal diet used in prefeeding period) and 7%, 14%, 21%, and 28% of defatted rice bran (DFRB) (as feed basis) substituted equivalent corn, respectively.

The basal diet was formulated according to the Feeding Standard of Swine 60 to 90 kg Standard of Meat-fat Type Growing-finishing Pig (NY/T 65-2004). The details of chemical composition and nutrition level of the experimental diets were shown in our previous study (15).

At the beginning of the experiment design, according to the “unique difference principle,” we not only used DFRB to replace corn to form fiber differences in each group but also slightly adjusted the content of wheat bran, soybean meal, and soybean oil in each group to make the calculated values of crude protein, amino acid, and metabolic energy (ME) close to the same. The analyzed chemical compositions of the DFRB and corn were shown in our previous study (15). All animals were healthy and did not receive any antibiotics during the whole experimental period.

### Sampling and measurements.

All pigs were slaughtered at the end of the experiment (day 28), and all pigs were fasted for 12 h before slaughter. After slaughter, the complete intestine was taken out immediately. After peeling off the mesentery on the surface of the large intestine, the weight of the intestine with contents was measured, and the length of the large intestine and cecum under natural placement was measured using a soft ruler. Subsequently, cecum samples with the size of about 3 cm × 4 cm were cut using a sterile scalpel and quickly stored in 4% paraformaldehyde for intestinal morphology observation. At the same time, the cecal tissue was washed with sterile saline to wash away intestinal contents on the mucosal surface (57). Then, the cecal mucosal microbial samples were scraped with sterile glass slides, loaded into a 2-mL sterile cryopreservation tube, and immediately stored in liquid nitrogen for metagenome sequencing.

Previous results showed that diet containing 19.10% TDF did not damage the fiber apparent digestibility and increased the cecal microbial diversity and the abundance of several fiber-degrading bacteria of pigs. However, when fed with diet containing 24.11% TDF, the fiber apparent digestibility of pigs decreased, and microbial changes disappeared (15). To analyze the characteristics of microbiota at species level and cecal epithelial structure of Suhuai pigs with the increasing of dietary fiber, therefore, the present study carried out further work with these three treatments: control group, treatment II, and treatment IV.

### Histomorphological investigations.

The evaluated morphometric indices were crypt depth, mucous thickness, submucosa thickness, muscularis thickness, and wall thickness (58, 59). Morphometric analyses were performed on 10-well oriented 10 crypts chosen from cecum (60). These morphometric indices were measured using a Nikon ECLIPSE 80i light microscope with a computer-assisted morphometric system (Nikon Corporation, Tokyo, Japan).

### Goblet cell staining.

The samples for goblet cell staining were prepared in accordance with the procedures for the cecal morphology analysis. The combined AB-PAS stain technique was then employed to measure the cecal goblet cell density (61). In particular, deparaffinized and rehydrated sections were stained with 1% Alcian Blue solution (Alcian Blue in 3% acetic acid solution), gently washed in double-distilled H_2_O for 10 min, oxidized in 1% periodic acid solution for 15 min, rinsed twice with double-distilled H_2_O for 10 min, then placed in periodic acid-Schiff solution for 30 min, and rinsed with running water for 5 min. Goblet cells were counted in 12-well oriented crypts per group, using the Nikon ECLIPSE 80i light microscope (Nikon Corporation, Tokyo, Japan). Goblet cell density was calculated as the goblet cell count divided by the corresponding crypt depth, averaged, and expressed as goblet cell number per 100 μm of crypt depth (62).

### Immunohistochemistry.

Ki-67 immunohistochemistry staining was used to determine cecal mucosal crypt cell proliferation (63). First, the slides were treated with an antigen retrieval process. The slides cannot be dried to prevent excessive evaporation of buffer solution during the process. After the slices were incubated with 3% hydrogen peroxide solution at room temperature and kept out of the light for 25 min to quench endogenous peroxidase, the slides were incubated with primary antibody (proliferation marker protein Ki-67; Wuhan Servicebio Biotechnology Co., Ltd., Hubei, China; 1:300) diluted in phosphate-buffered saline (PBS) overnight at 4°C. Then, the slides were incubated with the biotinylated goat anti-rabbit secondary antibody for 50 min. Finally, di-amino-benzidine (DAB) chromogenic reagent was used to control the desired stain intensity. Twelve random crypts in each slice were analyzed. The total number of crypt cells and the number of Ki-67-positive cells (brown cells) in each crypt were counted to calculate the percentage of proliferative cells.

We then detected the percentage of apoptotic cells in the cecum of pigs using terminal deoxynucleotidyl transferase-mediated dUTP nick end labeling (TUNEL) staining. The slides were rehydrated as follows: soaking the slices in xylene for 10 min; replacing the xylene; soaking for another 10 min; washing with PBS three times; and soaking with 100% ethanol, 95% ethanol, 85% ethanol, and 70% ethanol, in order. Then, the slides were incubated with 10× proteinase K for 15 to 20 min at 37°C. The slides were placed in the H_2_O_2_ solution (3% in methanol) for 20 min at 25°C to deactivate endogenous peroxides, followed by washing with PBS three times. After that, the slides were incubated with terminal deoxynucleotidyl transferase (TDT) for 60 min at 37°C, followed by washing with PBS three times. The slides were incubated with horseradish peroxidase-conjugated streptavidin solution (streptavidin-HRP) for 30 min at 37°C, followed by washing with PBS three times. After that, the slides were incubated with DAB solution and hematoxylin solution in turn. The number of TUNEL-positive cells (brown cells) for every 200 cecal epithelium cells in 10 random mucosal areas per slide were counted to calculate the percentage of apoptotic cells.

### Shotgun metagenomic sequencing.

Total genomic DNA was extracted from cecal mucosal samples using the E.Z.N.A. soil DNA kit (Omega Bio-tek, Norcross, GA, USA) according to the manufacturer’s instructions. The concentration and purity of extracted DNA was determined with TBS-380 and NanoDrop2000, respectively. DNA extract quality was checked on 1% agarose gel.

DNA extract was fragmented to an average size of about 400 bp using Covaris M220 (Gene Company Limited, Shanghai, China) for paired-end library construction. Paired-end library was constructed using NEXTFLEX Rapid DNA-Seq (Bios Scientific, Austin, TX, USA). Adapters containing the full complement of sequencing primer hybridization sites were ligated to the blunt end of fragments. Paired-end sequencing was performed on Illumina Hiseq Xten (Illumina Inc., San Diego, CA, USA) at Majorbio Bio-Pharm Technology Co., Ltd. (Shanghai, China) using HiSeq X reagent kits according to the manufacturer’s instructions (www.illumina.com).

The data were analyzed on the free online platform of Majorbio Cloud Platform (www.majorbio.com). The paired-end Illumina reads were trimmed of adaptors, and low-quality reads (length < 50 bp or with a quality value < 20 or having *N* bases) were removed by fastp (64) (https://github.com/OpenGene/fastp, version 0.20.0). Reads were aligned to the pig genome by BWA (65) (http://bio-bwa.sourceforge.net, version 0.7.9a), and any hits associated with the reads and their mated reads were removed.

### Bioinformatics and statistical analysis.

Comparing the differences of morphometric indices, number of goblet cells, and immunohistochemistry indices between each of treatment groups with the control group was conducted by using one-way analysis of variance (ANOVA) followed by a least-significant difference (LSD) *post hoc* test using SPSS 20.0. Polynomial contrasts were conducted to determine the linear effects of inclusion level of dietary fiber on the above indexes using SPSS 20.0. For comparing the data of relative abundance of microbial genes and functions, a Wilcoxon rank-sum test was performed for pairwise comparisons. Spearman’s correlation analysis (two-tailed test) was conducted to detect the relationships between intestinal indexes and microbial microbiota, and the relationships were visualized using Origin 2021. The bacterial taxa information of 16S rRNA gene sequencing in Fig. 4, the data of SCFAs in Table S3, and the amount of digested crude protein (CP), ether extract (EE), IDF, SDF, and TDF in Fig. 9 are from our previous study (15). Statistical significance was defined as *P < *0.05.

### Ethics approval.

All experimental animals were handled according to Guidelines for the Care and Use of Laboratory Animals prepared by the Institutional Animal Welfare and Ethics Committee of Nanjing Agricultural University, Nanjing, China.

### Data availability.

Data of metagenomic sequencing associated with this project have been deposited in the NCBI Short Read Archive database (accession number PRJNA781281).
