# Supplementary material for: Increased Proportion of Fiber-Degrading Microbes and Enhanced Cecum Development Jointly Promote Host To Digest Appropriate High-Fiber Diets
Source: mSystems. 2022 Dec 13;8(1):e00937-22. doi: 10.1128/msystems.00937-22 (PMC9948726; doi:10.1128/msystems.00937-22)
Supplement: TABLE S1 [file msystems.00937-22-s0003.docx]

| SCFAs | Cecal mucosal morphology | Correlation coefficient | *P* value |
| --- | --- | --- | --- |
| butyrate | Mucous thinckness, μm | -0.150 | 0.700 |
|  | Submucosa thickness, μm | 0.083 | 0.831 |
|  | Muscularis thickness, μm | 0.233 | 0.546 |
|  | Intestinal wall thickness, μm | 0.083 | 0.831 |
|  | Goblet cells of cecam, n/100 μm | 0.633 | 0.067 |
| Total SCFAs | Mucous thinckness, μm | -0.376 | 0.332 |
|  | Submucosa thickness, μm | 0.167 | 0.668 |
|  | Muscularis thickness, μm | 0.500 | 0.170 |
|  | Intestinal wall thickness, μm | 0.100 | 0.798 |
|  | Goblet cells of cecam, n/100 μm | 0.583 | 0.099 |
