# Supplementary material for: Increased Proportion of Fiber-Degrading Microbes and Enhanced Cecum Development Jointly Promote Host To Digest Appropriate High-Fiber Diets
Source: mSystems. 2022 Dec 13;8(1):e00937-22. doi: 10.1128/msystems.00937-22 (PMC9948726; doi:10.1128/msystems.00937-22)
Supplement: TABLE S2 [file msystems.00937-22-s0005.docx]

| Amount of digested dietary fiber, g/d | Cecal mucosal morphology | Correlation coefficient | *P* value |
| --- | --- | --- | --- |
| IDF | Mucous thinckness, μm | 0.018 | 0.938 |
|  | Submucosa thickness, μm | 0.517 | 0.016 |
|  | Muscularis thickness, μm | 0.409 | 0.066 |
|  | Intestinal wall thickness, μm | 0.519 | 0.016 |
|  | Goblet cells of cecam, n/100 μm | 0.600 | 0.004 |
| SDF | Mucous thinckness, μm | -0.035 | 0.880 |
|  | Submucosa thickness, μm | 0.560 | 0.008 |
|  | Muscularis thickness, μm | 0.470 | 0.032 |
|  | Intestinal wall thickness, μm | 0.564 | 0.008 |
|  | Goblet cells of cecam, n/100 μm | 0.690 | 0.001 |
| TDF | Mucous thinckness, μm | 0.018 | 0.938 |
|  | Submucosa thickness, μm | 0.517 | 0.016 |
|  | Muscularis thickness, μm | 0.409 | 0.066 |
|  | Intestinal wall thickness, μm | 0.519 | 0.016 |
|  | Goblet cells of cecam, n/100 μm | 0.600 | 0.004 |

Note: IDF: Insoluble dietary fiber. SDF: soluble dietary fiber. TDF: total dietary fiber.
