# Supplementary figures and images for: Increased Proportion of Fiber-Degrading Microbes and Enhanced Cecum Development Jointly Promote Host To Digest Appropriate High-Fiber Diets
Source: mSystems. 2022 Dec 13;8(1):e00937-22. doi: 10.1128/msystems.00937-22 (PMC9948726; doi:10.1128/msystems.00937-22)

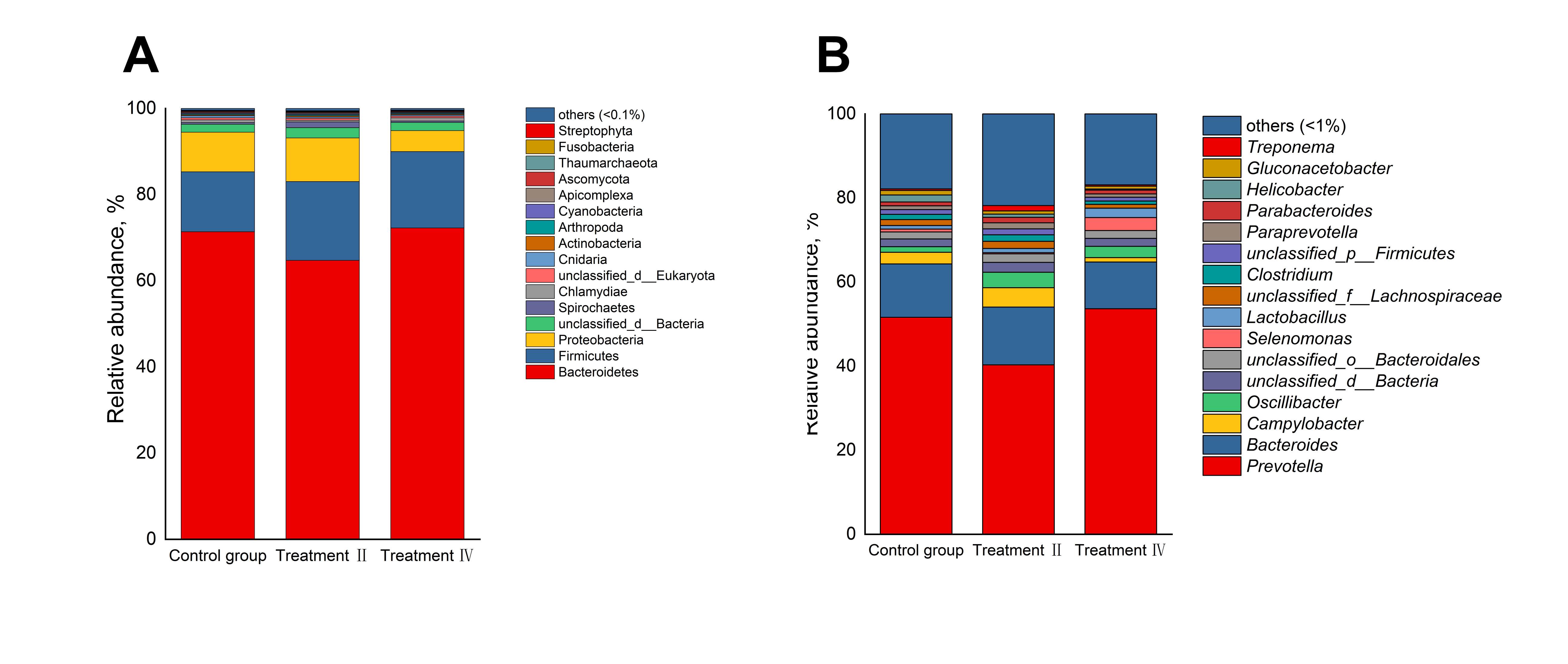

Supplement: FIG S1 [file msystems.00937-22-s0001.tif]

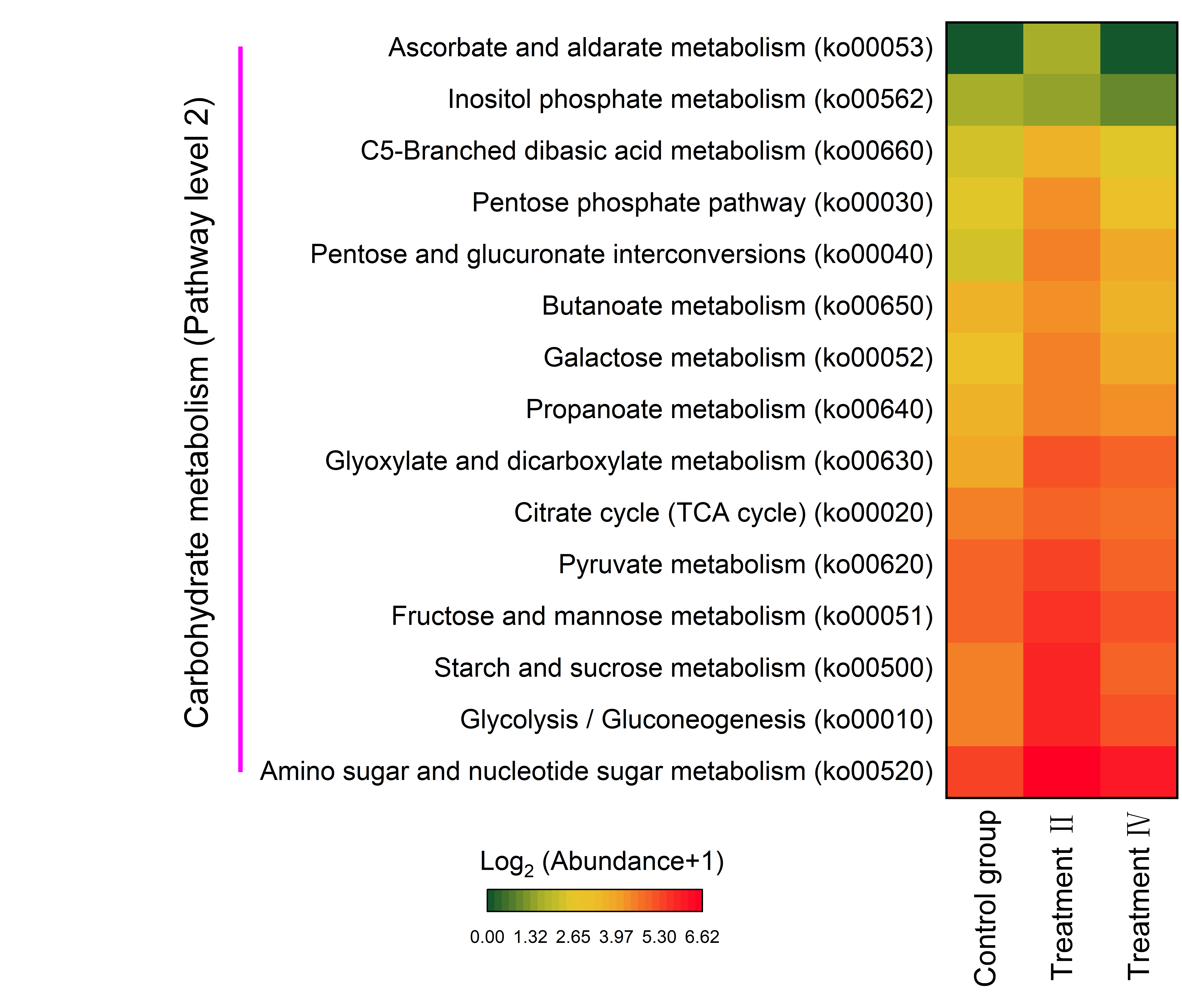

Supplement: FIG S3 [file msystems.00937-22-s0004.tif]
