# Supplementary material for: Increased Proportion of Fiber-Degrading Microbes and Enhanced Cecum Development Jointly Promote Host To Digest Appropriate High-Fiber Diets
Source: mSystems. 2022 Dec 13;8(1):e00937-22. doi: 10.1128/msystems.00937-22 (PMC9948726; doi:10.1128/msystems.00937-22)
Supplement: TABLE S3 [file msystems.00937-22-s0006.docx]

| Samples | Raw reads | Raw base (bp) | Contigs | Contigs bases (bp) | N50 (bp) | N90 (bp) | Max (bp) | Min (bp) |
| --- | --- | --- | --- | --- | --- | --- | --- | --- |
| Con_1 | 69732782 | 12576345000 | 161494 | 115456526 | 848 | 347 | 20721 | 300 |
| Con_2 | 74864724 | 11569509300 | 161301 | 120483754 | 904 | 352 | 19140 | 300 |
| Con_3 | 72810458 | 14619541500 | 169040 | 124253549 | 887 | 352 | 10726 | 300 |
| Con_4 | 82818578 | 11888738100 | 175598 | 147482069 | 1050 | 370 | 47370 | 300 |
| Con_5 | 83983246 | 13521132900 | 171460 | 144686324 | 1087 | 371 | 27016 | 300 |
| Con_6 | 90140886 | 10596034200 | 192000 | 167779755 | 1136 | 374 | 44103 | 300 |
| Con_7 | 81399944 | 11862939300 | 163607 | 136408169 | 1075 | 367 | 18500 | 300 |
| Tre_2_1 | 76703306 | 11147059500 | 174654 | 138105518 | 943 | 376 | 35244 | 300 |
| Tre_2_2 | 77130062 | 14867236200 | 182382 | 142960495 | 981 | 358 | 21256 | 300 |
| Tre_2_3 | 77539342 | 12209991600 | 152805 | 127166199 | 1006 | 397 | 17157 | 300 |
| Tre_2_4 | 97463610 | 10921568700 | 180207 | 169205795 | 1248 | 402 | 42332 | 300 |
| Tre_2_5 | 99114908 | 9181827000 | 160684 | 158916962 | 1333 | 424 | 25431 | 300 |
| Tre_2_6 | 83842300 | 10314000600 | 185625 | 152051315 | 1050 | 363 | 23972 | 300 |
| Tre_2_7 | 79258254 | 10459917300 | 164964 | 134603108 | 1032 | 364 | 19358 | 300 |
| Tre_4_1 | 80798872 | 10088054400 | 172958 | 146305303 | 1063 | 375 | 40847 | 300 |
| Tre_4_2 | 67253696 | 11505495900 | 156569 | 117003360 | 899 | 355 | 12868 | 300 |
| Tre_4_3 | 70640228 | 12119830800 | 167320 | 127499758 | 950 | 360 | 16397 | 300 |
| Tre_4_4 | 68760004 | 12597486900 | 202127 | 150833114 | 917 | 351 | 28454 | 300 |
| Tre_4_5 | 79086262 | 11630901300 | 172290 | 142968915 | 1059 | 369 | 43390 | 300 |
| Tre_4_6 | 74313730 | 11229708600 | 185025 | 138003093 | 947 | 356 | 23148 | 300 |
| Tre_4_7 | 61212180 | 12422786700 | 160820 | 108644414 | 815 | 343 | 17692 | 300 |

Note: The first column are the names of 21 samples
