# Supplementary material for: Increased Proportion of Fiber-Degrading Microbes and Enhanced Cecum Development Jointly Promote Host To Digest Appropriate High-Fiber Diets
Source: mSystems. 2022 Dec 13;8(1):e00937-22. doi: 10.1128/msystems.00937-22 (PMC9948726; doi:10.1128/msystems.00937-22)
Supplement: TABLE S4 [file msystems.00937-22-s0007.docx]

| Items | Phylum | Class | Order | Family | Genus | Species |
| --- | --- | --- | --- | --- | --- | --- |
| Shotgun metagenomic sequencing analysis | 55 | 102 | 192 | 346 | 1069 | 3703 |
| 16S rRNA gene sequencing analysis | 24 | 42 | 80 | 136 | 321 | 603 |
