# Supplementary material for: Increased Proportion of Fiber-Degrading Microbes and Enhanced Cecum Development Jointly Promote Host To Digest Appropriate High-Fiber Diets
Source: mSystems. 2022 Dec 13;8(1):e00937-22. doi: 10.1128/msystems.00937-22 (PMC9948726; doi:10.1128/msystems.00937-22)
Supplement: TABLE S5 [file msystems.00937-22-s0008.docx]

| Name | Tre_Ⅱ^1^-Mean (%) | Sd (%) | Tre^2^_Ⅳ-Mean (%) | Sd (%) | *P* value |
| --- | --- | --- | --- | --- | --- |
| *s__Methanosarcina_barkeri* | 0.0044 | 0.0031 | 0.0050 | 0.0035 | 0.125 |
| *s__Firmicutes_bacterium_CAG:345* | 0.0040 | 0.0010 | 0.0017 | 0.0023 | 0.072 |
| *s__uncultured_bacterium_fosmid_pJB154B8_contig_I* | 0.0134 | 0.0049 | 0.0063 | 0.0043 | 0.021 |
| *s__Coprobacter_secundus* | 0.2274 | 0.0611 | 0.1437 | 0.0805 | 0.055 |
| *s__Peptoclostridium_difficile* | 0.1117 | 0.0340 | 0.0665 | 0.0312 | 0.030 |
| *s__unclassified_f__Porphyromonadaceae* | 0.1099 | 0.0284 | 0.0563 | 0.0414 | 0.041 |
| *s__Tannerella_sp._CAG:51* | 0.0704 | 0.0189 | 0.0383 | 0.0210 | 0.030 |
| *s__Bacteroidales_bacterium_Barb6XT* | 0.0381 | 0.0087 | 0.0286 | 0.0071 | 0.022 |
| *s__Alistipes_finegoldii* | 0.0356 | 0.0079 | 0.0276 | 0.0100 | 0.201 |
| *s__Dysgonomonas_sp._BGC7* | 0.0367 | 0.0125 | 0.0146 | 0.0098 | 0.007 |
| *s__Clostridium_sp._ATCC_BAA-442* | 0.0206 | 0.0063 | 0.0191 | 0.0208 | 0.201 |
| *s__Bacteroidales_bacterium_KA00251* | 0.0181 | 0.0042 | 0.0115 | 0.0062 | 0.074 |
| *s__Phaeodactylibacter_xiamenensis* | 0.0034 | 0.0022 | 0.0028 | 0.0006 | 0.080 |
| *s__uncultured_bacterium_fosmid_pJB102C1* | 0.0126 | 0.0068 | 0.0037 | 0.0026 | 0.021 |
| *s__uncultured_rumen_bacterium* | 0.0151 | 0.0066 | 0.0086 | 0.0092 | 0.074 |
| *s__Fibrobacter_succinogenes* | 0.0523 | 0.0098 | 0.0367 | 0.0238 | 0.074 |
| *s__Dysgonomonas_capnocytophagoides* | 0.0531 | 0.0086 | 0.0341 | 0.0174 | 0.055 |
| *s__Porphyromonas_asaccharolytica* | 0.0266 | 0.0082 | 0.0217 | 0.0124 | 0.371 |
| *s__Chryseobacterium_palustre* | 0.0033 | 0.0028 | 0.0010 | 0.0016 | 0.089 |
| *s__Polaribacter_sp._Hel_I_88* | 0.0019 | 0.0016 | 0.0011 | 0.0017 | 0.333 |
| *s__Denitrobacterium_detoxificans* | 0.0033 | 0.0020 | 0.0014 | 0.0021 | 0.054 |
| *s__Clostridium_nexile_CAG:348* | 0.0105 | 0.0060 | 0.0084 | 0.0100 | 0.250 |
| *s__Porphyromonas_sp._COT-290_OH860* | 0.0095 | 0.0045 | 0.0031 | 0.0034 | 0.021 |
| *s__Mesoflavibacter_zeaxanthinifaciens* | 0.0037 | 0.0017 | 0.0020 | 0.0019 | 0.097 |
| *s__Firmicutes_bacterium_CAG:83* | 0.0132 | 0.0109 | 0.0097 | 0.0102 | 0.307 |
| *s__Dysgonomonas_mossii* | 0.0633 | 0.0171 | 0.0409 | 0.0177 | 0.011 |
| *s__Bacteroides_coprocola_CAG:162* | 0.0393 | 0.0107 | 0.0257 | 0.0097 | 0.041 |
| *s__Dysgonomonas_sp._HGC4* | 0.0410 | 0.0139 | 0.0227 | 0.0112 | 0.041 |
| *s__Bacteroides_propionicifaciens* | 0.0362 | 0.0135 | 0.0317 | 0.0110 | 0.798 |
| *s__Veillonella_montpellierensis* | 0.0333 | 0.0089 | 0.0281 | 0.0132 | 0.160 |
| *s__uncultured_bacterium_fosmid_pJB89E1* | 0.0313 | 0.0119 | 0.0214 | 0.0093 | 0.125 |
| *s__Butyrivibrio_sp._AE3003* | 0.0284 | 0.0067 | 0.0145 | 0.0132 | 0.074 |
| *s__Sanguibacteroides_justesenii* | 0.0214 | 0.0056 | 0.0173 | 0.0071 | 0.250 |
| *s__uncultured_bacterium_Contigcl_1748* | 0.0206 | 0.0052 | 0.0171 | 0.0148 | 0.201 |
| *s__Porphyromonas_levii* | 0.0156 | 0.0048 | 0.0128 | 0.0071 | 0.201 |
| *s__Clostridium_sp._CAG:264* | 0.0139 | 0.0046 | 0.0091 | 0.0068 | 0.201 |
| *s__Porphyromonas_crevioricanis* | 0.0128 | 0.0051 | 0.0163 | 0.0059 | 0.250 |
| *s__Algoriphagus_terrigena* | 0.0036 | 0.0024 | 0.0010 | 0.0009 | 0.041 |
| *s__Firmicutes_bacterium_CAG:449* | 0.0026 | 0.0020 | 0.0004 | 0.0007 | 0.010 |
| *s__Solitalea_canadensis* | 0.0086 | 0.0044 | 0.0020 | 0.0020 | 0.011 |
| *s__Hymenobacter_norwichensis* | 0.0071 | 0.0032 | 0.0089 | 0.0089 | 1.000 |
| *s__Alistipes_finegoldii_CAG:68* | 0.0066 | 0.0047 | 0.0017 | 0.0011 | 0.021 |
| *s__Empedobacter_brevis* | 0.0042 | 0.0032 | 0.0028 | 0.0017 | 0.523 |
| *s__Proteiniphilum_sp._51_7* | 0.0121 | 0.0073 | 0.0047 | 0.0049 | 0.097 |
| *s__uncultured_bacterium_URE4* | 0.0092 | 0.0073 | 0.0061 | 0.0045 | 0.523 |
| *s__Cellulophaga_lytica* | 0.0050 | 0.0049 | 0.0021 | 0.0022 | 0.125 |
| *s__Bacteroides_barnesiae* | 0.2285 | 0.0485 | 0.2080 | 0.0832 | 0.201 |
| *s__Eubacterium_plexicaudatum* | 0.1371 | 0.0240 | 0.0809 | 0.0421 | 0.021 |
| *s__Parabacteroides_merdae* | 0.1316 | 0.0273 | 0.0805 | 0.0254 | 0.011 |
| *s__Bacteroides_sp._CAG:770* | 0.1324 | 0.0469 | 0.0976 | 0.0134 | 0.097 |
| *s__Coprobacter_fastidiosus* | 0.1212 | 0.0388 | 0.0684 | 0.0364 | 0.030 |
| *s__Paludibacter_jiangxiensis* | 0.0912 | 0.0454 | 0.0566 | 0.0138 | 0.041 |
| *s__Intestinimonas_butyriciproducens* | 0.0965 | 0.0497 | 0.0588 | 0.0445 | 0.125 |
| *s__Bacteroides_sp._CAG:20* | 0.0632 | 0.0191 | 0.0350 | 0.0173 | 0.015 |
| *s__Oscillibacter_sp._KLE_1745* | 0.0585 | 0.0356 | 0.0477 | 0.0487 | 0.307 |
| *s__Dorea_formicigenerans* | 0.0464 | 0.0187 | 0.0319 | 0.0186 | 0.201 |
| *s__Odoribacter_sp._CAG:788* | 0.0411 | 0.0168 | 0.0263 | 0.0102 | 0.160 |
| *s__Ruminococcaceae_bacterium_cv2* | 0.0369 | 0.0120 | 0.0267 | 0.0226 | 0.250 |
| *s__[Eubacterium]_hallii* | 0.0306 | 0.0142 | 0.0149 | 0.0133 | 0.030 |
| *s__Treponema_denticola* | 0.0278 | 0.0148 | 0.0409 | 0.0566 | 0.443 |
| *s__Porphyromonas_gingivicanis* | 0.0249 | 0.0079 | 0.0123 | 0.0068 | 0.015 |
| *s__Alistipes_sp._CAG:268* | 0.0265 | 0.0224 | 0.0152 | 0.0075 | 0.307 |
| *s__[Eubacterium]_siraeum* | 0.0169 | 0.0063 | 0.0143 | 0.0167 | 0.097 |
| *s__Eggerthella_sp._CAG:1427* | 0.0127 | 0.0037 | 0.0077 | 0.0042 | 0.055 |
| *s__Ruminococcus_sp._CAG:379* | 0.0095 | 0.0045 | 0.0046 | 0.0035 | 0.055 |
| *s__Youngiibacter_fragilis* | 0.0080 | 0.0025 | 0.0040 | 0.0026 | 0.030 |

Note: The difference between treatment Ⅱ and treatment Ⅳ were tested by Wilcoxon rank-sum test. ^1^ Tre-Ⅱ, treatment Ⅱ.^2^ Tre-Ⅳ, treatment Ⅳ. SD, standard deviation.
