# Supplementary material for: Increased Proportion of Fiber-Degrading Microbes and Enhanced Cecum Development Jointly Promote Host To Digest Appropriate High-Fiber Diets
Source: mSystems. 2022 Dec 13;8(1):e00937-22. doi: 10.1128/msystems.00937-22 (PMC9948726; doi:10.1128/msystems.00937-22)
Supplement: TABLE S6 [file msystems.00937-22-s0009.docx]

| Name | Con-Mean (%) | Sd (%) | Tre Ⅳ-Mean (%) | Tre_4-Sd (%) | Pvalue |
| --- | --- | --- | --- | --- | --- |
| *s__Schistosoma_japonicum* | 0.0000 | 0.0000 | 0.0732 | 0.1480 | 0.004 |
| *s__Chlamydia_psittaci* | 0.0042 | 0.0041 | 0.0516 | 0.0567 | 0.007 |
| *s__Anaerophaga_thermohalophila* | 0.0096 | 0.0071 | 0.0433 | 0.0345 | 0.005 |
| *s__Prevotella_sp._HJM029* | 0.0230 | 0.0076 | 0.0407 | 0.0098 | 0.007 |
| *s__Mannheimia_haemolytica* | 0.0371 | 0.0067 | 0.0543 | 0.0098 | 0.005 |
| *s__Lactobacillus_equi* | 0.0000 | 0.0000 | 0.0116 | 0.0287 | 0.004 |
| *s__Centipeda_periodontii* | 0.0010 | 0.0011 | 0.0126 | 0.0088 | 0.002 |
| *s__Shigella_sonnei* | 0.0051 | 0.0028 | 0.0159 | 0.0056 | 0.003 |
| *s__bacterium_SIT8* | 0.0054 | 0.0031 | 0.0147 | 0.0063 | 0.007 |
| *s__Schistosoma_mansoni* | 0.0000 | 0.0000 | 0.0065 | 0.0057 | 0.004 |
| *s__Fermentimonas_caenicola* | 0.0051 | 0.0021 | 0.0114 | 0.0033 | 0.005 |
| *s__Bacteroidetes_bacterium_OLB9* | 0.0000 | 0.0000 | 0.0040 | 0.0041 | 0.004 |
| *s__Croceibacter_atlanticus* | 0.0002 | 0.0004 | 0.0038 | 0.0029 | 0.004 |
| *s__Helicobacter_felis* | 0.0000 | 0.0000 | 0.0030 | 0.0022 | 0.004 |
| *s__Enterococcus_pallens* | 0.0051 | 0.0041 | 0.0006 | 0.0012 | 0.006 |
| *s__Helicobacter_mustelae* | 0.0056 | 0.0063 | 0.0000 | 0.0000 | 0.004 |
| *s__Campylobacter_subantarcticus* | 0.0111 | 0.0126 | 0.0007 | 0.0012 | 0.004 |
| *s__Campylobacter_sp._FOBRC14* | 0.0129 | 0.0186 | 0.0007 | 0.0007 | 0.007 |
| *s__Campylobacter_cuniculorum* | 0.0174 | 0.0204 | 0.0029 | 0.0027 | 0.007 |
| *s__Helicobacter_pullorum* | 0.0762 | 0.0586 | 0.0106 | 0.0071 | 0.005 |
