# Supplementary material for: Increased Proportion of Fiber-Degrading Microbes and Enhanced Cecum Development Jointly Promote Host To Digest Appropriate High-Fiber Diets
Source: mSystems. 2022 Dec 13;8(1):e00937-22. doi: 10.1128/msystems.00937-22 (PMC9948726; doi:10.1128/msystems.00937-22)
Supplement: TABLE S7 [file msystems.00937-22-s0010.docx]

| Name | Tre_2-Mean (%) | Tre_2-Sd (%) | Tre_4-Mean (%) | Tre_4-Sd (%) | Pvalue |
| --- | --- | --- | --- | --- | --- |
| *s__Selenomonas_bovis* | 0.0243 | 0.0213 | 0.2585 | 0.2538 | 0.005 |
| *s__Prevotella_denticola* | 0.2014 | 0.0753 | 0.3372 | 0.0878 | 0.007 |
| *s__Prevotella_histicola* | 0.1144 | 0.0379 | 0.2437 | 0.1129 | 0.007 |
| *s__Lactobacillus_salivarius* | 0.0011 | 0.0023 | 0.0312 | 0.0360 | 0.007 |
| *s__Megasphaera_cerevisiae* | 0.0044 | 0.0043 | 0.0163 | 0.0092 | 0.007 |
| *s__Lactobacillus_equi* | 0.0000 | 0.0000 | 0.0118 | 0.0291 | 0.004 |
| *s__Ruminococcus_sp._SR1/5* | 0.0029 | 0.0027 | 0.0091 | 0.0041 | 0.005 |
| *s__Shigella_sonnei* | 0.0001 | 0.0003 | 0.0039 | 0.0048 | 0.006 |
| *s__Clostridium_arbusti* | 0.0002 | 0.0002 | 0.0039 | 0.0048 | 0.005 |
| *s__Mannheimia_haemolytica* | 0.0000 | 0.0000 | 0.0033 | 0.0062 | 0.004 |
| *s__Helicobacter_felis* | 0.0003 | 0.0009 | 0.0070 | 0.0082 | 0.002 |
| *s__Chryseobacterium_sp._YR561* | 0.0017 | 0.0013 | 0.0001 | 0.0002 | 0.003 |
| *s__Sulfurimonas_autotrophica* | 0.0036 | 0.0042 | 0.0000 | 0.0001 | 0.006 |
| *s__Enterococcus_cecorum* | 0.0077 | 0.0050 | 0.0010 | 0.0011 | 0.005 |
| *s__Campylobacter_lanienae* | 0.0069 | 0.0103 | 0.0002 | 0.0004 | 0.002 |
| *s__unclassified_g__Treponema* | 0.0082 | 0.0070 | 0.0011 | 0.0017 | 0.007 |
| *s__Brachyspira_hampsonii* | 0.0076 | 0.0071 | 0.0001 | 0.0002 | 0.002 |
| *s__Acidiphilium_multivorum* | 0.0082 | 0.0090 | 0.0005 | 0.0008 | 0.004 |
| *s__[Clostridium]_saccharolyticum* | 0.0147 | 0.0045 | 0.0062 | 0.0047 | 0.007 |
| *s__Clostridium_disporicum* | 0.0241 | 0.0052 | 0.0134 | 0.0067 | 0.007 |
| *s__Nitratiruptor_sp._SB155-2* | 0.0113 | 0.0156 | 0.0004 | 0.0010 | 0.004 |
| *s__Alistipes_indistinctus* | 0.0270 | 0.0042 | 0.0160 | 0.0048 | 0.003 |
| *s__Butyrivibrio_sp._MC2021* | 0.0206 | 0.0068 | 0.0088 | 0.0054 | 0.007 |
| *s__Campylobacter_subantarcticus* | 0.0138 | 0.0093 | 0.0008 | 0.0012 | 0.002 |
| *s__Lachnospiraceae_bacterium_6_1_37FAA* | 0.0162 | 0.0086 | 0.0030 | 0.0023 | 0.003 |
| *s__Campylobacter_sp._MIT_97-5078* | 0.0152 | 0.0187 | 0.0018 | 0.0042 | 0.009 |
| *s__Campylobacter_volucris* | 0.0186 | 0.0271 | 0.0010 | 0.0014 | 0.007 |
| *s__Campylobacter_cuniculorum* | 0.0251 | 0.0291 | 0.0030 | 0.0029 | 0.005 |
| *s__Campylobacter_gracilis* | 0.0340 | 0.0275 | 0.0035 | 0.0038 | 0.003 |
| *s__Helicobacter_apodemus* | 0.0383 | 0.0397 | 0.0059 | 0.0058 | 0.003 |
| *s__Campylobacter_sp._FOBRC14* | 0.0340 | 0.0426 | 0.0007 | 0.0007 | 0.002 |
| *s__Campylobacter_rectus* | 0.0389 | 0.0310 | 0.0021 | 0.0009 | 0.002 |
| *s__Campylobacter_hominis* | 0.0419 | 0.0430 | 0.0020 | 0.0016 | 0.002 |
| *s__Campylobacter_corcagiensis* | 0.0476 | 0.0580 | 0.0016 | 0.0015 | 0.002 |
| *s__Helicobacter_sp._MIT_05-5294* | 0.0531 | 0.0641 | 0.0068 | 0.0041 | 0.005 |
| *s__Campylobacter_upsaliensis* | 0.0527 | 0.0654 | 0.0045 | 0.0029 | 0.002 |
| *s__Campylobacter_ureolyticus* | 0.0535 | 0.0628 | 0.0027 | 0.0017 | 0.002 |
| *s__Campylobacter_curvus* | 0.0565 | 0.0686 | 0.0010 | 0.0014 | 0.002 |
| *s__Helicobacter_pullorum* | 0.0681 | 0.0683 | 0.0110 | 0.0075 | 0.007 |
| *s__Ruminococcus_sp._CAG:108* | 0.0622 | 0.1355 | 0.0027 | 0.0031 | 0.003 |
| *s__Campylobacter_sputorum* | 0.0729 | 0.0814 | 0.0013 | 0.0012 | 0.002 |
| *s__Campylobacter_showae* | 0.0796 | 0.0880 | 0.0080 | 0.0057 | 0.002 |
| *s__Campylobacter_coli* | 0.1110 | 0.1464 | 0.0198 | 0.0136 | 0.007 |
| *s__Campylobacter_mucosalis* | 0.1095 | 0.1625 | 0.0052 | 0.0019 | 0.002 |
| *s__Campylobacter_concisus* | 0.1230 | 0.1269 | 0.0067 | 0.0039 | 0.002 |
| *s__unclassified_g__Campylobacter* | 0.1504 | 0.1669 | 0.0099 | 0.0041 | 0.002 |
| *s__Campylobacter_hyointestinalis* | 0.9437 | 1.3010 | 0.0193 | 0.0158 | 0.002 |
| *s__Campylobacter_iguaniorum* | 1.2360 | 1.5350 | 0.0404 | 0.0216 | 0.002 |
| *s__Campylobacter_fetus* | 3.1750 | 4.0360 | 0.0863 | 0.0497 | 0.002 |
